# Spatial prediction of dynamic interactions in rats

**DOI:** 10.1371/journal.pone.0319101

**Published:** 2025-02-25

**Authors:** Tereza Dvorakova, Veronika Lobellova, Paloma Manubens, Abel Sanchez-Jimenez, Jose Antonio Villacorta-Atienza, Ales Stuchlik, David Levcik

**Affiliations:** 1 Laboratory of Neurophysiology of Memory, Institute of Physiology of the Czech Academy of Sciences, Prague, Czech Republic; 2 Department of Biodiversity, Ecology, and Evolution, Unit of Biomathematics, Faculty of Biology, Complutense University of Madrid, Madrid, Spain; Universidad de Guadalajara, MEXICO

## Abstract

Animals and humans receive the most critical information from parts of the environment that are immediately inaccessible and highly dynamic. The brain must effectively process potential interactions between elements in such an environment to make appropriate decisions in critical situations. We trained male Long-Evans rats to discriminate static and dynamic spatial stimuli and to generalize novel dynamic spatial stimuli displayed on an inaccessible computer screen. We provide behavioral evidence indicating that rats encode dynamic visuospatial situations by constructing internal static representations that capture meaningful future interactions between objects. These observations support previous findings in humans that such internal static representations can encapsulate relevant spatiotemporal information of dynamic environments. This mechanism would allow animals and humans to process complex time-changing situations neatly.

## Introduction

Spatial cognition is essential for most animal species, including humans, as it allows them to locate resources efficiently, avoid obstacles and predators, and navigate complex environments. This ability is allowed by the so-called cognitive map – a mental representation of the surrounding environment [1]. Such a map includes, among other information, spatial relationships between objects and places and their relative distances and directions [2,3].

Static environments, where the locations of objects and landmarks remain constant, allow individuals to form stable cognitive maps based on their experiences and interactions with that environment. Laboratory spatial tasks for rodents typically require navigation to target places within stable environments [4,5]. However, the real world is inherently unstable. Spatial cognition must operate in dynamic environments, where multiple objects may change locations with variable velocities and directions, demanding continuous real-time updating and coordination of information from different sources. Despite the abundant wealth of knowledge regarding spatial cognition, there remains a significant gap in understanding the processing of dynamic spatial information.

Animals must represent not only their position within an environment but also the positions of other objects in their surroundings. Rodent spatial tasks often involve approaching and contacting other objects to discriminate their positions [6,7]. Therefore, in most experimental paradigms, items of interest are placed within accessible parts of a familiar environment so that animals can associate previously visited positions with objects’ locations [8]. However, in natural settings, important spatial information often comes from distal (visual) parts of the environment without direct access [9]. Distant landmarks, such as trees or the position of the sun, serve as essential cues for navigation. The ability to process and represent such inaccessible stimuli is critical for efficient spatial cognition. Thus, incorporating out-of-reach visual stimuli in behavioral experiments allows for a more comprehensive understanding of how animals form representations of their environments.

How rodents perceive visual stimuli became more widely studied with the advent of behavioral tasks that utilized computer screens and virtual environments. Such an experimental setup enables complete automation and precise control over presented stimuli and introduces analogous versions of human tasks for rodents. Rats can discriminate positions of visual objects displayed on a distant computer screen [10,11], a process that depends on the hippocampus [12]. The hippocampus is well-established as critical for navigation, both in static environments [2] and dynamic scenarios [13]. Beyond spatial navigation, the hippocampus plays a pivotal role in episodic memory, which involves the ability to recall specific events, including their spatial and temporal contexts [14,15]. This capacity is closely tied to working memory, as the hippocampus supports the integration and short-term retention of spatial and temporal information necessary for forming episodic-like memories in rodents [16,17]. Despite these insights, how dynamic objects and their potential interactions are encoded, particularly in inaccessible spaces that reflect more common and natural situations, remains poorly understood. This gap in understanding forms the basis for our investigation, which aims to explore how rats process and represent dynamic visuospatial stimuli.

**Fig 1:**
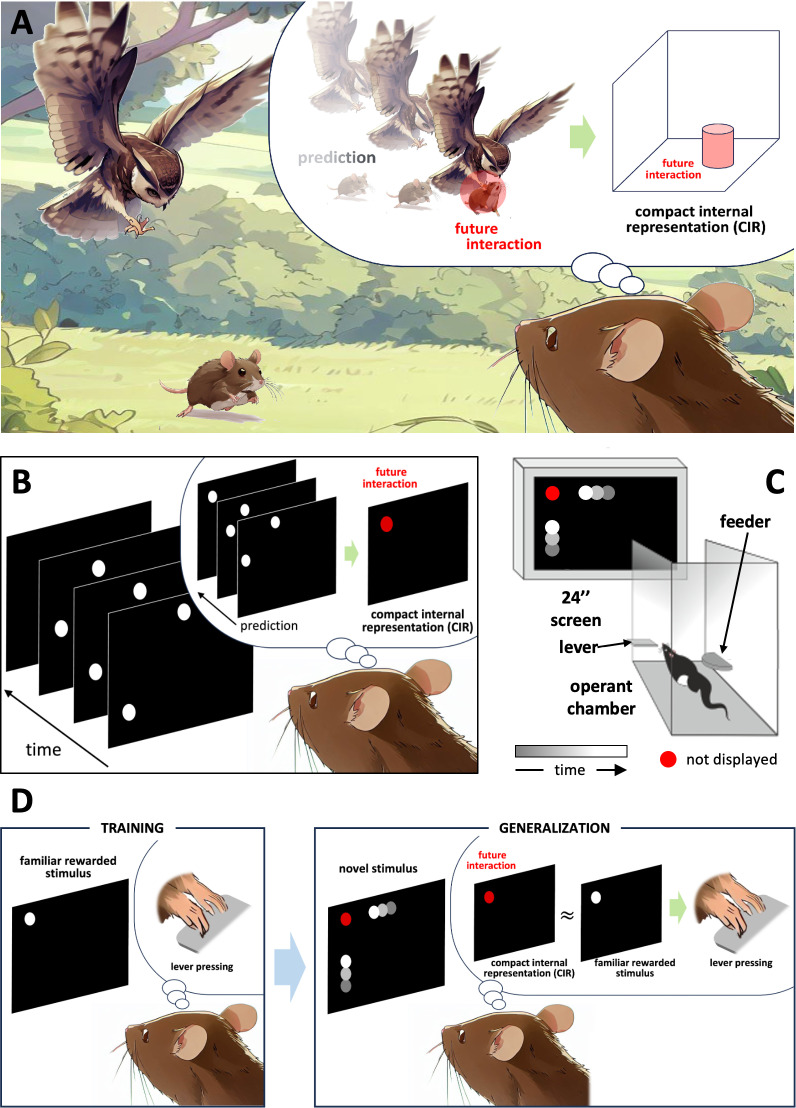
Prediction of a dynamic interaction. (A) Time compaction theory proposes that a dynamic scenario is internally represented by statically mapping the predicted future interactions. This compact internal representation (CIR) enables efficient learning and memorization of dynamic situations [19], allowing real-time decision-making, a primary requirement for survival. (B) Visuospatial prediction of future interaction of dynamic stimuli presented on the distant computer screen during the visuospatial discrimination (VSD) task. (C) Scheme of the behavioral apparatus used for the VSD task with an example of dynamic stimulus. The time grayscale indicates the direction of movement of the circles (from bottom to top and from right to left in the figure), which disappear before colliding. The red circle denotes the position of the future collision between the circles, which was not displayed to the rat. (D) Experimental hypothesis. After training, the rat associates the particular position of a static circle with a reward obtained after pressing the lever (left). Then, when a novel dynamic stimulus is presented with two moving circles that would collide at the position of the static circle in the familiar rewarded stimulus, the rat should tend to press the lever since the CIR of the novel stimulus is similar to the familiar rewarded stimulus (right; circle grayscale and stimulus red circle as in panel C).

A mechanism called time compaction could underlie the processing of distal dynamic situations [18]. This mechanism introduces a paradoxical concept, suggesting that the brain codifies time by removing it, representing a dynamic situation as future critical interactions among elements encapsulated in a static map. According to the time compaction hypothesis, a dynamic situation is neurally represented as a compact internal representation (CIR, Fig 1A and 1B). The CIR functions as a static map, spatially encoding all possible interactions between individual elements in a given environment, effectively removing time from the dynamic situation while retaining all the necessary information for navigation [19]. The existence of time compaction in humans has been recently demonstrated [20]. Evidence for CIR in rats provides an opportunity for comparative studies which are crucial for identifying common mechanisms underlying cognitive processes across species.

We designed our study to understand the mechanisms of the internal representation of our spatiotemporal world, specifically the processing of dynamic visuospatial scenes. We 1) tested the compact internal representation in rats and 2) compared the discrimination of static and complementary dynamic visuospatial stimuli. We 1) hypothesized that rats would utilize a compact internal representation (CIR) to process dynamic visuospatial stimuli, encoding the predicted future interactions between objects in a static format and 2) that rats would demonstrate superior discrimination performance for dynamic visuospatial stimuli compared to static stimuli, reflecting a cognitive prioritization of dynamic scenarios critical for real-time decision-making and survival. Our results show that rats can internally represent dynamic visuospatial scenes as static representations capturing future interactions between objects in the environment and discriminating dynamic visuospatial stimuli faster and more precisely than static ones.

## Materials and methods

### Animals

The experimental subjects were outbred male Long-Evans rats (N = 6 for the static version of the task, N = 8 for the dynamic version) three months old at the beginning of the experiment. The rats were obtained from the Animal Facility of the Institute of Physiology CAS (Prague, Czech Republic; for the static version of the task) or the Charles River Laboratories breeding colony (Calco, Italy; for the dynamic version of the task) and were housed in pairs. The animal room was kept at a constant temperature of 21 °C and a 12-hour light/dark cycle. All handling and experiments were performed during the light phase of the day. Rats had free access to water but were limited to food to be maintained at 90% of their free-feeding weight. After transportation from the breeding colony, the rats were left to acclimatize for ten days and then handled for 5 mins every day for five days before the beginning of the behavioral training. All the methods were performed in accordance with relevant institutional guidelines and regulations. All animal treatments were approved by the Local Animal Care Committee (50/2017) and complied with the Animal Protection Code of the Czech Republic and the European Community Council directive (2010/63/EC). The experiments were conducted, and all methods were reported in accordance with ARRIVE guidelines. After the completion of experiments, we utilized isoflurane overdose as the method of sacrifice to ensure a humane approach. Maximum efforts were made to minimize the suffering of animals.

### Behavioral apparatus

The apparatus consisted of an operant chamber (length x width x height: 24 cm × 14 cm × 36 cm) with a lever and a feeder, a 24” LCD monitor (1920 × 1080 pixels, 16:9 aspect ratio) placed 37 cm in front of the chamber, and a computer (Fig 1C, see [21] for further apparatus details). The computer displayed the stimuli on the screen, registered lever presses, and activated the feeder. In the case of a rewarded lever press, a 45 mg chocolate-flavored pellet (Bio-Serv, USA) was delivered from the feeder to the operant chamber. The stimuli were displayed by software written in C++. During the experiments, rats were trained and tested in two identical apparatuses. Each rat was randomly assigned to a particular apparatus and always trained and tested in the same apparatus.

### Visuospatial discrimination (VSD) task - static version

**Fig 2:**
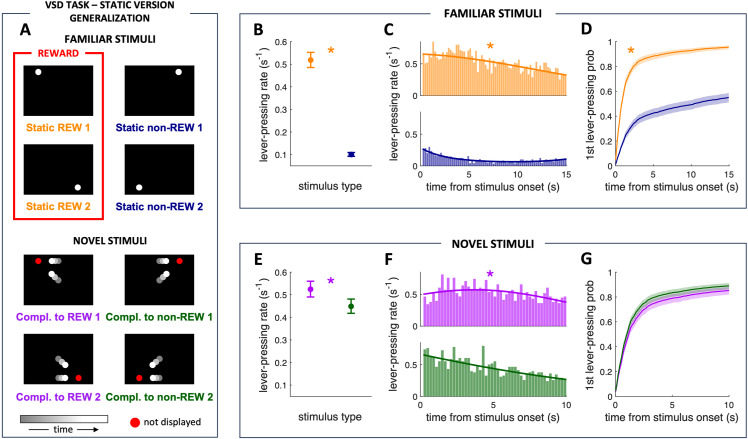
Compact internal representation of future interactions in rats. (A) VSD task - static version: Generalization test. We used two familiar rewarded static stimuli (Static REW1 and Static REW2, in red rectangle) and two familiar non-rewarded static stimuli (Static non-REW1 and Static non-REW2) in this generalization test. In addition, we presented the rats with four novel stimuli pseudorandomly displayed between the presentations of the four familiar training stimuli. The novel dynamic stimuli had the location of the predicted collision identical to the static positions of familiar training stimuli (red circle, not displayed to the rats): Compl. to REW1 to Static REW1, Compl. to REW2 to Static REW2, Compl. to non-REW1 to Static non-REW1, Compl. to non-REW2 to Static non-REW2. No novel stimulus was rewarded. The time grayscale denotes the direction of movement of the circles in the novel stimuli, so they move from gray to white positions. The rats discriminated between rewarded and non-rewarded familiar static object positions. (B) Mean rate of pressing the lever during the presentations of the two types of familiar stimuli in the generalization test, with the solid lines depicting the regression curves that model the temporal structure of the lever-pressing rate (odds ratio (OR) = 6.53). (C) Lever-pressing rate distribution throughout the two types of familiar stimuli duration (histograms binned at 200 ms). (D) Probability of the first lever press throughout the duration of the two types of familiar stimuli after the stimulus onset. The orange asterisk marks significant differences (p < 0.0001). The rats pressed the lever more often during the novel dynamic stimuli in which two circles approached the familiar static rewarded positions. (E) Main rate of pressing the lever duringthe presentations of the two types of novel stimuli in the generalization test (odds ratio (OR) = 1.24). (F) Lever-pressing rate distribution throughout the two types of novel stimuli duration (histograms binned at 200 ms), with the regression curve modeling the temporal structure given by the solid line. (G) Probability of the first lever press throughout the duration of the two types of novel stimuli after the stimulus onset. The purple asterisk marks significant differences (p < 0.0001). Familiar stimuli lasted 15 sec. Novel dynamic stimuli lasted 10 sec to prevent learning during the session that these stimuli were not rewarded. Data in panels B, D, E, and G are shown as means ± SEM. The rat and session were introduced as random effect (grouping) factors and time (trial) and stimulus type (rewarded/non-rewarded) as fixed effect factors (the same for subsequent GLMM analyses; see Materials and methods).

In the static version of the VSD task, we trained the food-deprived rats (N = 6) to discriminate by lever-pressing two particular static rewarded positions of a white circle (d = 5.85 cm; Fig 2A, upper left, marked in a red rectangle; Static REW1, Static REW2, where REW denotes reward) and two static non-rewarded positions of the same circle (Fig 2A, upper right; Static non-REW1, Static non-REW2, where non-REW denotes non-reward). One-half of the rats (N = 3) were trained with rewarded and non-rewarded stimulus combinations shown in Fig 2A, upper. The other half of rats (N = 3) were trained with the swapped combination of stimuli, i.e., the rewarded stimulus combination in the first half of the rats was the non-rewarded combination in the second half of the rats and vice versa. We used this design to exclude potential bias for a particular stimulus. For our analyses, we merged data from both halves of the rats as we did not observe any prominent sub-group-related differences in the discrimination performance of the trained rats.

We progressively used five training configurations with increased rat performance demands. We gradually shortened the presentation of the stimuli from 90 s to 15 s and changed the reinforcement schedule from continuous to variable ratio 3. Individual visual stimuli lasted 15 s and were separated by 3 s blank screen periods in the final (fifth) configuration of the behavioral training. The increased demands on attention (shorter stimulus duration and variable ratio 3) prevent the rats from using the feedback of the apparatus (reward delivery) to solve the task. The rats were typically able to obtain up to four reward pellets during each 15 s rewarded presentation.

Before starting the behavioral training, the food-deprived rats were pre-trained to press the lever in the operant chamber to obtain a food pellet. Then, we trained the rats once a day in the VSD task, five days a week, until they reached the performance criteria in the final training configuration of the task.

In the initial training configuration, individual stimuli were displayed for 90 s and were separated by 5 s blank screen pauses. We trained the rats under the continuous reinforcement schedule. Both rewarded stimuli were displayed three times, and both non-rewarded stimuli six times during the initial training sessions, which lasted 28 min 30 s. The sequence of the stimuli was pseudorandom to prevent the rats from using alternative strategies to solve the task. In addition, the same stimulus was never displayed twice in a row, and neither were two rewarded stimuli. As the non-rewarded stimuli were presented twice as often as the rewarded ones, the chance level performance was 33.3%. Following our previous studies using a similar experimental setup [12,21], we set the threshold for reliable performance in the task to 60% of correct responses.

When the rats reliably responded to the rewarded stimuli (60% or more correct presses for at least four out of five previous training sessions), we gradually shortened the presentation of the stimuli from 90 s to 15 s, and we changed the schedule of reinforcement from continuous to the variable ratio 3 in the final training configuration. Therefore, both rewarded stimuli were displayed 18 times, and both non-rewarded stimuli 36 times during the final training sessions. Blank pauses between stimuli lasted 3 s, and the final training session lasted 32 min 24 s. The parameters of all training configurations are described in S1 Table.

The pseudorandom sequences of particular stimuli repeated during the training sessions of the static version of the VSD task are described below. These sequences were repeated six times during a single final training configuration session. We used a different sequence for each apparatus to prevent the rats from synchronizing their responses based on the sound of the feeder.

VSD task – static version, first apparatus - training sessions: Static REW 1, Static non-REW 1, Static REW 2, Static non-REW 2, Static non-REW 1, Static REW 2, Static non-REW 2, Static non-REW 1, Static non-REW 2, Static REW 1, Static non-REW 2, Static REW 2, Static non-REW 1, Static non-REW 2, Static REW 1, Static non-REW 1, Static non-REW 2, Static non-REW 1.

VSD task – static version, second apparatus - training sessions: Static REW 1, Static non-REW 1, Static non-REW 2, Static non-REW 1, Static REW 2, Static non-REW 2, Static non-REW 1, Static REW 2, Static non-REW 2, Static REW 1, Static non-REW 2, Static non-REW 1, Static non-REW 2, Static REW 2, Static non-REW 1, Static non-REW 2, Static REW 1, Static non-REW 1.

### VSD task - static version: Generalization test

Once the rats mastered the static version of the VSD task, we conducted a generalization test. We introduced four novel dynamic spatial stimuli to examine the rats’ ability to apply their acquired knowledge of the static positions of the familiar white circle, whether rewarded or non-rewarded, to discern unfamiliar dynamic stimuli implying future collision at those positions. The hypothesis, illustrated in Fig 1D, was that if the animals could discriminate the novel dynamic stimuli in the generalization phase based on the critical static spatial information – the position of the predicted collision (Fig 2A, lower; red circles), they would press the lever more often during the novel dynamic stimuli consisted of two circles that moved in a direction placing the anticipated collision point where the position of the static circle was in the familiar Static REW stimuli than during the novel dynamic stimuli consisted of two circles that moved in a direction placing the anticipated collision point at the position of the static circle in the familiar Static non-REW stimuli, i.e., during Compl. to REW (denoting Complementary to reward) stimuli than during Compl. to non-REW (denoting Complementary to non-reward) stimuli. Lever presses during Compl. to REW stimuli were not rewarded, just as lever presses during Compl. to non-REW stimuli were not.

The novel dynamic stimuli consisted of two white circles (d = 5.85 cm) moving in the direction of the circles in the familiar static stimuli. However, both moving circles disappeared in the middle of their trajectories. Each dynamic circle approach lasted for 1 second, with the next approach starting immediately after the previous one disappeared in the middle of the circle trajectories. Therefore, the complete novel dynamic stimulus in the generalization test, lasting 10 s, consisted of 10 repetitions of a particular approach. We displayed the four novel stimuli pseudorandomly between the presentations of the four familiar training stimuli during the generalization test. Lever presses during novel dynamic stimuli were not rewarded for preventing learning and stimulus bias during the test.

Each novel dynamic stimulus (Compl. to REW 1, Compl. to REW 2, Compl. to non-REW 1, Compl. to non-REW 2; Fig 2A, lower) was displayed 12 times during the generalization test (six consecutive repetitions of the pseudorandom sequence of stimuli – see below). Individual presentations lasted only 10 s to decrease the likelihood of learning that the novel stimuli are non-rewarded. Both familiar rewarded stimuli (Static REW 1, Static REW 2) were displayed 18 times, and both familiar non-rewarded stimuli (Static non-REW 1, Static non-REW 2) were displayed 36 times during the generalization test. The blank pauses between individual stimuli lasted 3 or 5 s (pseudorandom distribution), and one complete generalization test took 45 min 24 s. The pseudorandom sequence of stimuli was the same for both subgroups, and only one rat was tested each time in the experimental room. The sequence is described below and was repeated six times during the generalization test.

In the novel dynamic stimuli, the upper/lower circles were moving at 6.23 cm/s, and the circles that started in the center of the screen had a speed of 8.8 cm/s. The pseudorandom sequence of stimuli displayed during this generalization test is described below, and it was repeated six times during a single test session.

VSD task – static version, both apparatuses - generalization tests: Static REW 1, Static non-REW 2, Static non-REW 1, Compl. to REW 1, Static non-REW 2, Compl. to non-REW 1, Static REW 2, Static non-REW 1, Static REW 1, Static non-REW 1, Compl. to non-REW 2, Static non-REW 2, Compl. to REW 2, Static non-REW 1, Static REW 2, Static non-REW 2, Compl. to non-REW 2, Static non-REW 1, Compl. to non-REW 1, Static REW 2, Static non-REW 2, Static non-REW 2, Compl. to REW 1, Static REW 1, Static non-REW 1, Compl. to REW 2.

We performed the generalization test two times. Five standard training sessions separated both tests to eliminate the effect of the first generalization test on the rats’ performance in the second test.

### VSD task - dynamic version

**Fig 3:**
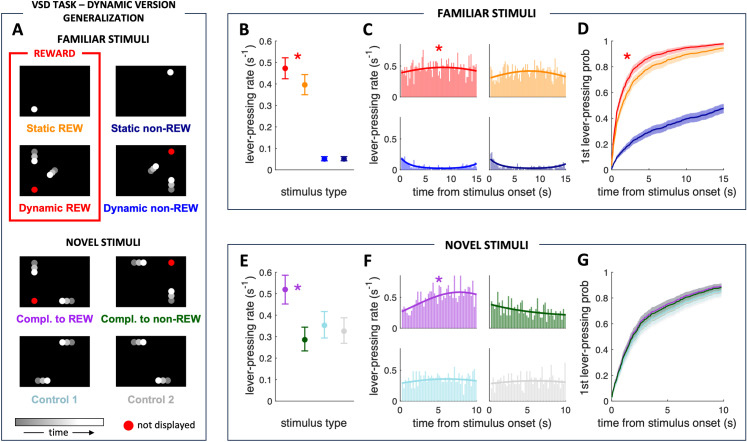
Supporting evidence of the compact internal representation of future interactions in rats. (A) VSD task - static version: Generalization test. During this generalization test, we presented the rat with four novel dynamic stimuli pseudorandomly displayed between the presentations of the four familiar training stimuli (shown in panel A, upper). One novel stimulus shared the location of the predicted collision with the Dynamic REW stimulus (Compl. to REW), another with the Dynamic non-REW stimulus (Compl. to non-REW), and two more stimuli had no predicted collision point (Control 1 and Control 2). The rats pressed the lever more often during the rewarded dynamic stimulus than the static one when novel non-rewarded dynamic stimuli were introduced. (B) Mean rate of pressing the lever during the presentations of individual familiar stimuli in the generalization test (odds ratio (OR) = 1.37). (C) Lever-pressing rate distribution throughout individual familiar stimuli duration (histograms binned at 200 ms and solid lines as in Fig 2). (D) Probability of the first lever press throughout individual familiar stimuli duration after the stimulus onset. The red asterisk marks significant differences (p < 0.0001) between the Dynamic REW stimulus and all other familiar stimuli; further statistical differences are described in the text. The rats pressed the lever with equal probability during the novel dynamic stimulus that shares the spatial information with rewarded familiar stimuli and during the rewarded familiar dynamic stimulus, and more often than during other novel stimuli. (E) Mean rate of pressing the lever during the presentations of individual novel stimuli in the generalization test (odds ratios: Compl. to REW vs. Compl. to non-REW OR = 2.6, Compl. to REW vs. Control 1 OR = 1.95, Compl. to REW vs. Control 2 OR = 2.2). (F) Lever-pressing rate distribution throughout individual novel stimuli duration (histograms binned at 200 ms and solid lines as in Fig 2). (G) Probability of the first lever press throughout individual novel stimuli duration after the stimulus onset. The purple asterisk marks significant differences (p < 0.0001) between the Compl. to REW stimulus and all other novel stimuli; further statistical differences are described in the text. Familiar stimuli lasted 15 sec. Novel dynamic stimuli lasted 10 sec to prevent learning during the session that these stimuli were not rewarded. Data in panels B, D, E, and G are shown as means ± SEM.

In the dynamic version of the VSD task, we trained a different group of food-deprived rats (N = 8) to discriminate a particular static rewarded position of a circle displayed on a distant computer screen and a complementary dynamic rewarded stimulus consisting of two circles that approached but did not reach the same static position (VSD task - dynamic version, Fig 3A). All aspects of the behavioral training and experimental design were the same as for the static version of the VSD task described above. The only difference was the set of used stimuli. The primary purpose of the dynamic version of the VSD task was to compare the discrimination of static and complementary dynamic visuospatial stimuli.

We used two types of visual stimuli in the dynamic version of the VSD task: static and dynamic. The static stimuli were a white circle (d = 5.85 cm) in the screen’s bottom-left or top-right (Fig 3A, upper). The two complementary dynamic stimuli consisted of two such circles moving in the direction of the circles in the static stimuli, implying future collision at those positions, but with both moving circles disappearing in the middle of their trajectories. The starting position of the first moving circle was the top left or bottom right part of the screen, respectively, and the second moving circle’s starting position was the screen’s center (Fig 3A, lower). Each dynamic circle approach lasted for 1 second, with the next approach starting immediately after the previous one disappeared in the middle of the circle trajectories. Therefore, the complete dynamic stimulus in the final configuration of the behavioral training, lasting 15 s, consisted of 15 repetitions of a particular approach. The laterally positioned circle was moving at 12.45 cm/s, and the circle that started in the center of the screen had a speed of 8.8 cm/s.

Of these four stimuli - two static and two dynamic - one static and the complementary dynamic were rewarded (Fig 3A, left, marked in a red rectangle; Static REW, Dynamic REW, where REW denotes reward), and the other static and its complementary dynamic version were non-rewarded (Fig 3A, right; Static non-REW, Dynamic non-REW, where non-REW denotes non-reward). One-half of the rats (N = 4) were trained with rewarded and non-rewarded stimulus combinations shown in Fig 3A, and the other half (N = 4) were trained with the swapped combination of stimuli. The pseudorandom sequences of particular stimuli repeated during the training sessions of the dynamic version of the VSD task are described below.

VSD task – dynamic version, first apparatus - training sessions: Static REW, Static non-REW, Dynamic REW, Dynamic non-REW, Static non-REW, Dynamic REW, Dynamic non-REW, Static non-REW, Dynamic non-REW, Static REW, Dynamic non-REW, Dynamic REW, Static non-REW, Dynamic non-REW, Static REW, Static non-REW, Dynamic non-REW, Static non-REW.

VSD task – dynamic version, second apparatus - training sessions: Static REW, Static non-REW, Dynamic non-REW, Static non-REW, Dynamic REW, Dynamic non-REW, Static non-REW, Dynamic REW, Dynamic non-REW, Static REW, Dynamic non-REW, Static non-REW, Dynamic non-REW, Dynamic REW, Static non-REW, Dynamic non-REW, Static REW, Static non-REW.

### VSD task - dynamic version: Generalization test

We performed another generalization test after the rats mastered the VSD task’s dynamic version. The design of this generalization test was identical to the one described for the static version of the task except for the set of presented stimuli.

During this generalization test, we displayed four novel non-rewarded dynamic stimuli pseudorandomly between the presentations of the four familiar training stimuli. Among the four novel non-rewarded dynamic stimuli comprising two moving circles, two stimuli shared the collision position with the previously discriminated dynamic stimuli. Specifically, one stimulus aligned with the Dynamic REW stimulus (Compl. to REW, denoting Complementary to reward), while the other aligned with the Dynamic non-REW stimulus (Compl. to non-REW, denoting Complementary to non-reward). The remaining two dynamic stimuli, without the predicted collision point, were included as control stimuli to assess the novelty effect (Control 1, Control 2) (Fig 3A, lower). Lever presses during Compl. to REW stimulus were not rewarded, just as lever presses during Compl. to non-REW, Control 1, and Control 2 stimulus were not. Single dynamic circle approaches again lasted 1 s, consisted of 10 repetitions of a particular approach, and the circles moved at 12.45 cm/s.

The hypothesis was that if the animals discriminated the dynamic spatial stimuli in the training phase based on the critical static information – the position of the predicted collision, they would press the lever more often during the novel dynamic stimulus that shares the position of the predicted collision with the previously discriminated Dynamic REW stimulus than during the novel dynamic stimulus that shares the position of the predicted collision with the previously discriminated Dynamic non-REW stimulus, i.e., during Compl. to REW stimulus than during Compl. to non-REW stimulus.

The pseudorandom sequence of stimuli displayed during this generalization test is described below, and it was repeated six times during a single test session.

VSD task – dynamic version, both apparatuses - generalization tests: Static REW, Dynamic non-REW, Static non-REW, Compl. to REW, Dynamic non-REW, Compl. to non-REW, Dynamic REW, Static non-REW, Static REW, Static non-REW, Control 2, Dynamic non-REW, Control 1, Static non-REW, Dynamic REW, Dynamic non-REW, Control 2, Static non-REW, Compl. to non-REW, Dynamic REW, Dynamic non-REW, Dynamic non-REW, Compl. to REW, Static REW, Static non-REW, Control 1.

We again performed the generalization test two times, and five standard training sessions separated both tests.

### Data analysis

For the static version of the VSD task, we analyzed exclusively the two generalization tests conducted after completed behavioral training. For the dynamic version of the VSD task, we analyzed data from both the generalization tests and the training sessions (examining learning dynamics and asymptotic performance).. Data from the generalization tests were used to analyze the possible transference of the acquired knowledge to discriminate a set of novel dynamic stimuli. We studied learning dynamics during the training sessions of the first training configuration and analyzed asymptotic performance in the last session of the final training configuration in the dynamic VSD task.

As described above, the rats were trained in both versions of the task to discriminate among four stimuli - two rewarded and two non-rewarded. The rewarded and non-rewarded stimuli were counterbalanced among rats to prevent bias. Counterbalancing involved systematically varying the spatial positions of stimuli (left/right, top/bottom) to control for potential spatial biases. During the generalization tests, four novel non-rewarded stimuli were introduced to assess the transference of learned associations. Stimulus duration was progressively shortened (from 90 sec to 15 sec) during training phases to increase task difficulty and promote attentional engagement. In the generalization phase, novel stimuli duration was further reduced to 10 sec to decrease the likelihood of learning that these stimuli were not rewarded.

Three main behavioral variables were analyzed to study rats’ performance during the presentation of individual stimuli: the mean probability of pressing the lever (mean number of lever presses per second), the probability distribution of lever-pressing during presentation periods (number of lever presses per ms within 200 ms bins over the time course of each presentation period), and the reaction time (time from the stimulus onset to the first lever press). Lever-pressing probability was analyzed utilizing a generalized linear mixed model (GLMM) with binomial distribution and logit link function (i.e., a logistic regression model). This model reveals what factors (stimulus type (i.e., static/dynamic, rewarded/non-rewarded), time and trial) significantly affect the lever-pressing probability when the effect is considered to vary for each rat and session (due to the lack of independence, the model includes a random intercept, taking the rat id and session, nested into rat id, as grouping factors). In contrast, a Cox Proportional Hazards model studied the reaction time. In all cases, we included stimulus type as a fixed effect factor. For the probability distribution analysis, the time from the stimulus onset (binned into 200 ms periods), its square, and its interactions with the stimulus type were also included as fixed effects factors. Analysis involving learning dynamics included the training duration, i.e., the number of sessions within each training configuration normalized for each rat by the maximum number of sessions that a rat underwent to reach the learning criteria of a particular configuration, as a fixed effect factor (both alone and in interaction with the stimulus type) and a random slope. For the sake of clarity, the probabilities of pressing the lever are also interpreted in terms of lever-pressing rates.

As rats were trained with only static stimuli in the static version of the VSD task, the stimulus type factor for models had two levels (Static REW/Static non-REW for the familiar stimuli, and Compl. to REW/Compl. to non-REW for the novel stimuli, respectively) instead of four levels as in the generalization session of the dynamic version of the VSD task.

A tentative full model including all factors and interactions was carried out in all regression models. Then, we selected variables by a stepwise backward procedure using *p*-values and AIC as criteria to exclude them until we reached a model with all significant factors. To assess differences between levels of significant categorical factors (stimuli types), type II Wald Chi-Square and Tukey posthoc tests with FDR correction were done. The level of significance was set to 0.05 in all cases. Data are shown as mean ± standard error of the mean (SEM). For a more detailed description of the studied variables, justification of the models used, and interpretation of the model’s coefficients, see S1 Appendix.

In order to clarify the effect size in the different experiments, on the one hand, we have graphically detailed the differences found, both on average and over time. On the other hand, we have reported the odds ratio and hazard ratio as quantitative measures of effect size. Under certain conditions (i.e., lower values of p) Odds Ratio (OR) is almost equal to relative risk so it could be interpreted as how much probability of success (pressing the lever in this case) is multiplied when comparing two levels of a categorical variable o when a continuous variable increases in one unit. In any case, values of OR greater than 1 indicate that the probability increases while values lower than 1 mean a decrease in the probability of success. Hazard Ratio (HR) indicates how much the probability of pressing the lever for the first time is multiplied at any time. This way, values of HR greater than 1 means that at any time this probability is greater and, for this reason, the measured event occurs earlier.

We carried out all analyses in R (version 4.1.3 [22]) under RStudio (version 2022.02.3 [23]). We used R packages lme4 [24], emmeans [25], survival [26], coxme [27], and ggplot2 [28].

## Results

### Generalization phase

We first present data from the generalization tests conducted on both static and dynamic VSD tasks. The results from the generalization phase of these experiments support the existence of CIRs (compact internal representations) in rats. These findings suggest that rats are capable of representing dynamic environments using static representations that encode future interactions of moving objects.

#### VSD task - static version.

On average, the rats took 82.7 (± 10.2 SEM) sessions to reach a stable target performance of > 60% of correct presses (see Materials and Methods for details) in the final configuration of the static version of the VSD task. Afterwards, we carried out the generalization tests.

The existence of a CIR of predicted interactions can be tested by the visuospatial discrimination of appropriate stimuli that share critical spatial information. The generalization phase of the static version of the VSD task utilizes novel dynamic stimuli (Fig 2A lower) with the point of future collision mirroring the familiar static stimuli positions from the learning phase (Fig 2A upper). According to the time compaction hypothesis, such complementary novel dynamic stimuli should be represented by CIRs that match the familiar static stimuli. Thus, we expected that rats previously trained to discriminate specific static stimuli would press the lever more frequently during novel dynamic stimuli when the locations of their future interactions correspond to the rewarded static stimuli positions.

#### Evidence of the CIR: the rats pressed the lever more often during the novel dynamic stimuli in which two circles approached the familiar static rewarded positions.

To precisely evaluate the differences between novel stimuli, we analyzed the non-familiar stimuli used in the generalization tests separately from the familiar stimuli. This approach aimed to eliminate any potential influence of novelty on the lever-pressing activity.

Both familiar static stimuli (REW and non-REW) were counterbalanced between the screen’s top/bottom and left/right sides. Regression models did not show any effect of these factors on the dynamics of lever-pressing behavior. Thus, to avoid any bias, they were combined so that the factor stimulus type only has two levels, REW and non-REW. Note that both generalization tests (see Data analysis) were combined for the analysis since the regression models again did not reveal any influence of these factors. During the generalization phase, the analysis revealed a higher lever-pressing rate during the Static REW stimuli than during the Static non-REW stimuli (p < 0.0001) (Fig 2B and 2C). Thus, the probability of lever-pressing during the presentation of the rewarded stimuli was 420% higher than while the non-rewarded stimuli were displayed (odds ratio (OR) = 6.53).

Visual stimulus type affected the time to the first lever press after the stimulus onset (Fig 2D), so the Cox Regression model showed a shorter time to the first operant response for the Static REW stimuli than for the Static non-REW stimuli (p < 0.0001). The hazard ratio (HR) for the Static REW stimuli was 2.82 times higher than for the Static non-REW stimuli, meaning that at any time after the onset of the stimulus, the probability that the lever has already been pressed was 180% higher for the Static REW stimuli than for the Static non-REW stimuli (median time of 1000 ms for the Static REW stimuli and 11640 ms for the Static non-REW stimuli, respectively).

The mean rate of pressing the lever was higher during the Compl. to REW stimuli than during Compl. to non-REW stimuli (p < 0.0001, Fig 2E). The generalized linear mixed model revealed that the interaction between the stimulus type and time was significant (p < 0.0001). This means that the temporal structure of the lever-pressing rate was different throughout the presentation of Compl. to REW and Compl. to non-REW stimuli (Fig 2F). In particular, the rats pressed the lever more often and in a more stable way during the novel dynamic stimuli that share the position of the predicted collision with the previously discriminated Static REW stimuli. The probability of lever-pressing during the presentation of the Compl. to REW stimuli were 17% higher than the Compl. to non-REW stimuli (odds ratio (OR) = 1.24).

There was no difference in the time the lever was pressed for the first time after the stimulus onset between the novel stimuli in the generalization tests (median times of 1280 ms and 800 ms for the Compl. to REW and Compl. to non-REW stimuli, respectively) (p = 0.30; Fig 2G).

#### VSD task - dynamic version.

To further assess the rats’ ability to represent the future interaction of two moving objects by their static coincidence, we conducted another generalization experiment where the shared information between familiar static and dynamic and novel moving generalization stimuli involves the location of their future interactions but in addition the dynamic generalized Compl. to REW/non-REW and familiar Dynamic REW/non-REW stimuli were partially visually overlapping (Fig 3A). As in the case of the static version of the VSD task, both generalization tests were combined for the analysis.

#### The rats pressed the lever more often during familiar rewarded dynamic than static stimulus.

During the generalization phase, the analysis concerning learned stimuli revealed higher lever-pressing rate during the Dynamic REW stimulus than during all other familiar stimuli (all p < 0.0001) and the Static REW stimulus than during both non-rewarded stimuli (both p < 0.0001). There was no difference between the two familiar non-rewarded stimuli (p = 0.97) (Fig 3B and 3C). The probability of lever-pressing during the presentation of the rewarded dynamic stimulus was 18% higher than while the rewarded static stimulus was displayed (odds ratio (OR) = 1.37).

Visual stimulus type again affected the time to the first lever press after the stimulus onset (p < 0.0001; Fig 3D). Tukey posthoc tests showed a shorter time to the first operant response for the Dynamic REW stimulus than for the three other stimuli (all p < 0.0001). There was a shorter time for the first lever press for the Static REW stimulus than for both non-rewarded stimuli (p < 0.0001). There were no differences between the two non-rewarded stimuli (p = 0.93). The hazard ratio (HR) for the Dynamic REW stimulus was 1.3 times higher than for the Static REW, meaning that at any time after the onset of the stimulus, the probability that the lever has already been pressed was 30% higher for the dynamic rewarded stimulus than for the static ones. In other words, during the Dynamic REW stimulus, the rats pressed the lever for the first time earlier than during the Static REW stimulus (median time of 1360 ms and 2000 ms for the Dynamic REW and Static REW stimulus, respectively; the median time was longer than the stimulus duration for both non-rewarded stimuli).

#### Supporting evidence of the CIR: the rats pressed the lever more often during the novel dynamic stimulus that shares the spatial information with rewarded familiar stimuli than during the rewarded familiar dynamic stimulus.

The existence of the CIR has been shown in the generalization phase of the static VSD task, but we wanted to provide further supporting evidence for the time compaction mechanism in rats and demonstrate that simple visual similarity between novel and familiar stimuli is not the only factor involved in the generalization of novel dynamic stimuli in our task. Thus, in addition to the common position of the static circle in Static REW and the future collision points in Dynamic REW and Compl. to REW stimuli, we included a shared visual feature (the vertical moving circle common for Dynamic REW and Compl. to REW stimuli, Fig 3A). Based on our previous experiments [22] and other studies [29,30], if the overlapping of visual information is the dominant mechanism driving the generalization process, we should expect a lower rate of lever-pressing during the novel Compl. to REW stimulus compared to the familiar Dynamic REW stimulus. However, the mean lever-pressing rate during the Compl. to REW stimulus was higher than the familiar Dynamic REW stimulus (p = 0.015; Fig 3B and 3E).

#### Supporting evidence of the CIR: the rats pressed the lever more often during the novel dynamic stimulus that shares the spatial information with rewarded familiar stimuli than during other novel stimuli.

The mean rate of pressing the lever was significantly higher during the Compl. to REW stimulus (i.e., the dynamic stimulus where the point of future collision coincides with that in the familiar dynamic stimulus (Dynamic REW) and with the position of the circle in the familiar static stimulus (Static REW)), than during other novel stimuli (all p < 0.0001, Fig 3E). Thus, the lever-pressing probability for the Compl. to REW stimulus is about 79% higher than during the Compl. to non-REW stimulus (odds ratio (OR) = 2.6) and 49% and 58% higher than during the Control 1 and Control 2 stimuli, respectively (odds ratio (OR) = 1.95, odds ratio (OR) = 2.2, respectively). The lever-pressing probability was significantly lower during the Compl. to non-REW stimulus than during the Control 1 stimulus (20% lower, p < 0.0001) and than during the Control 2 stimulus (10% lower, p = 0.029). Both control stimuli showed similar probabilities (p = 0.15).

Since the interaction between the stimulus type and time was significant (p < 0.0001), the probability of pressing the lever throughout the presentation of individual stimuli was different between the stimuli (Fig 3F). The rats pressed the lever more often during the novel dynamic stimulus that shares the position of the predicted collision with the previously discriminated Dynamic REW stimulus, i.e., the Compl. to REW stimulus, than during the other three novel stimuli (p < 0.0001). None of the control stimuli showed a significant dependence on time, neither the linear nor the quadratic component, while the Compl. to non-REW stimulus (the stimulus that shares the place of the predicted collision with the previously discriminated Dynamic non-REW stimulus) depended only on the linear component of time with a negative slope. Both linear and quadratic components of time had a significant effect (p < 0.0001 in both cases) on the probability of pressing the lever during the presentation of the Compl. to REW stimulus.

In contrast to the familiar stimuli, there was no difference between the time the rats pressed the lever for the first time after each stimulus onset among the novel stimuli in the generalization tests (p = 0.21; Fig 3G). The lever was pressed for the first time after the stimulus onset at similar times for all four stimuli (median times of 1920 ms, 1520 ms, 1860 ms, and 1960 ms for the Compl. to REW, Compl. to non-REW, Control 1, and Control 2 stimulus, respectively).

### Learning phase

In nature, threats and opportunities for survival are essentially dynamic. Thus, animals must primarily deal with dynamic scenarios requiring critical real-time decisions. Therefore, we hypothesize that dynamic stimuli are prioritized over static stimuli in central cognitive processes such as learning. To test this hypothesis, we analyzed the learning phase of the dynamic VSD task in which rats were trained to discriminate static and complementary dynamic visuospatial stimuli.

#### First learning configuration: faster learning and shorter reaction time of dynamic versus static stimulus discrimination.

**Fig 4:**
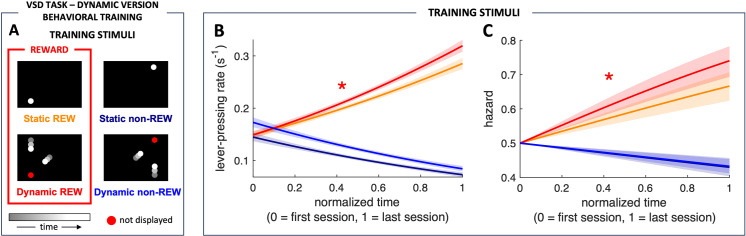
Faster learning and shorter reaction time of dynamic versus static stimulus discrimination. (A) VSD task - dynamic version. We presented the rats with two complementary rewarded stimuli – one static and one dynamic (Static REW and Dynamic REW, in red rectangle) and two complementary non-rewarded stimuli (Static non-REW and Dynamic non-REW). The static stimuli were stable white circles, and the complementary dynamic stimuli consisted of two circles moving toward the static circle location (red circle – predicted collision point; not displayed) but disappearing half of the way (gray-to-white color of the circles denote their movement direction, given by the time grayscale). (B) Lever-pressing rate for individual stimuli during the first training configuration (odds ratio (OR) = 1.18). (C) Hazard ratios of the first lever press after the stimulus onset for individual stimuli during the first training configuration. The red asterisk marks significant differences (p < 0.01 for panel B, **p** < 0.05 for panel C) between the Dynamic REW stimulus and all other stimuli. Further statistical differences are described in the text. Data in panels B and C are shown as means ± SEM (Dynamic-REW vs. Static-REW hazard ratio (HR) = 1.19).

We assessed rats’ learning performance by the probability of pressing the lever during the presentation of particular stimuli. The discriminated stimuli were one static and the complementary dynamic stimulus, in which the future collision area of the moving circles matches the position of the static stimulus (Fig 4A; see Materials and Methods). During the first training configuration, the probability of lever-pressing increased with the training progression for rewarded stimuli while decreasing for non-rewarded ones (p < 0.0001 for all four slopes). Although there were no differences between slopes of non-rewarded stimuli (p = 0.31), lever-pressing probability increased faster during Dynamic-REW than during Static-REW stimulus (p = 0.005), so at the end of the first training configuration, this probability was 30% higher for dynamic than for static rewarded stimulus (odds ratio (OR) = 1.18, Fig 4B). On the other hand, the hazard ratio analysis showed that the reaction time decreased with training progress for rewarded stimuli while increased for non-rewarded stimuli (p < 0.0001 for all comparisons of any rewarded stimulus with any non-rewarded stimulus) (Fig 4C). While the probability of pressing the lever for the first time at any time from the onset of the stimulus is 19% higher in Dynamic-REW compared with Static-REW (p = 0.029, HR = 1.19), there were no differences between non-rewarded stimuli (p = 0.64, HR = 1.09). These findings suggest faster learning of dynamic visual stimuli than similar static stimuli.

As individual rats underwent different numbers of sessions during discrete training configurations (S2 Table), we normalized the learning time for individual configurations to a scale from 0 (first session in the particular training configuration) to 1 (last session in the specific training configuration). Learning curves for all four stimuli were prominent only during the first configuration of the task (Fig 4B). During the subsequent configurations, in which we gradually shortened the presentation time of individual stimuli and increased the schedule of reinforcement, learning curves were not significant or selective for a particular stimulus (see S1 Fig, S2 Fig, S3 Fig, and S4 Fig; and S2 Appendix for complete results from configurations 2^nd^ – 5^th^). The reason for including these subsequent configurations was to increase the demands on attention during the task and prevent the rats from using the feedback of the apparatus (reward delivery) to solve the task. If we maintain a constant reinforcement schedule and keep the stimulus duration too long, the rats might continue to respond even after receiving a reward from accidentally pressing the lever during a rewarded stimulus. This could result in their responses not accurately reflecting their understanding of the task. Our previous studies have shown that an effective way to train the rats to master a similar task is by gradually shortening the stimulus duration and increasing the reinforcement schedule [17–19,21]. Otherwise, their performance does not improve, as a dramatic decrease in stimulus duration or a sudden increase in the reinforcement schedule is too demanding and does not allow the rats to adapt effectively to the increased task demands.

Out of the eight rats trained in the dynamic version of the VSD task, seven reached a stable target performance of > 60% of correct presses in the final configuration of the task. On average, the rats took 46 (± 2.4 SEM) sessions to be fully trained in the task (see S2 Table for more details).

See S3 Table for comprehensive statistical data for Figs 2–4.

## Discussion

Nature is essentially dynamic, and survival requires efficient visual information processing to make real-time critical decisions and cope with threats and demands. In this work, we present data obtained from behavioral experiments in rats supporting the involvement of the previously introduced time compaction mechanism in representing dynamic environments and faster processing of dynamic stimuli over static ones.

First, our results suggest that rats can effectively use the time compaction mechanism to internally represent dynamic spatial situations as static images capturing predicted object interactions. Second, we showed that rats discriminate dynamic visual objects faster and more precisely than static visual objects. This work supports the encoding of future interactions as an efficient mechanism of spatiotemporal cognition, suggesting it as an evolutionary invariant after similar phenomena are reported in humans [20] and bats [31]. Moreover, spatiotemporal processing is essential for episodic memory [32], and its impairment is a precursor to cognitive decline and has consequences for various neuropsychiatric illnesses, including dementia and schizophrenia [33–36]. Therefore, the obtained information could open new venues for creating innovative therapeutic strategies to combat cognitive deterioration. In addition, developing transparent and interpretable artificial navigation systems and robots with animal-like navigation abilities can also be aided by understanding the mechanics underlying spatiotemporal cognition in dynamic environments [37,38].

We designed unique behavioral experiments to test rats’ processing of static and dynamic objects outside their direct reach. The dynamic nature of objects and their inaccessibility are characteristics typical of natural conditions that were overlooked in previous studies. Furthermore, we presented the rats with static and dynamic stimuli that complemented their spatial aspects, allowing their direct comparison.

### Rats can use the time compaction mechanism to represent dynamic visuospatial environments

The mechanisms of processing complex dynamic environments are unknown. In pigeons, neurons that encode the time [39,40] or distance [41] to the collision of objects approaching the animal were discovered. However, to effectively navigate natural environments, animals must not process only potential encounters with looming objects but also interactions of and with other moving objects in their surroundings.

The generalization tests of our VSD task showed that the rats could transfer previously acquired knowledge and generalize novel visuospatial stimuli. They pressed the lever more often during the novel dynamic stimuli in which the two circles approached the familiar rewarded static positions or the position of predicted collision of circles in the familiar rewarded dynamic stimulus.

It was previously shown that rats could generalize static visual objects based on their spatial characteristics – specifically by estimating the distance between the familiar object and the novel object’s positions [10] or the degree of geometric similarity between objects displayed at the same location [22]. A mechanism called time compaction has been introduced to decipher how time-changing environments could be encoded in the brain [18]. The inclusion of the time dimension in processing dynamic situations introduces a significant amount of redundant information, which has the potential to hinder the speed and accuracy of neural processing of individual events. This might be problematic, especially under critical life-threatening circumstances. Therefore, it would be beneficial for complex dynamic environments to be internally represented simply, enabling faster processing. Time compaction proposes that the brain embeds time into space rather than directly encoding it. This way, spatial dynamic situations are internally processed as compact internal representations (CIRs) – static maps containing the possible interactions between elements in a given environment. Since the CIRs encode future interactions between the environmental elements, they relate to the subject’s ability to predict the future positions of moving objects [20]. Following the time compaction hypothesis and the obtained results, we propose that rats could predict the future positions of moving objects in our task, imagine their potential collisions, and use the time compaction mechanism to generalize the novel stimuli in the generalization tests.

Future subjects’ positions and movement directions are represented by distinct neural mechanisms nested in the hippocampus – a brain structure that plays a crucial role in spatial cognition. Several neuronal types have been discovered within the rodent hippocampus that give rise to a mental representation of the environment, known as a cognitive map [1]. These include the hippocampal place cells that encode the animal’s position in space [42]. Theta phase precession, a phenomenon that ties together the firing of place cells with the typical hippocampal theta rhythm, has been shown to predict movement direction [43]. Moreover, place cells have been found to fire in time-compressed sequences that predict one’s future trajectory during the periods of sharp wave ripples, the high-frequency oscillatory patterns presented in the mammalian hippocampus [44,45]. This way, future animals’ trajectories are pre-played in the brain within a few hundred milliseconds, while the real path lasts a few seconds. Additionally, the hippocampus also exhibits evidence of backward replay, where sequences of place-cell activity associated with past trajectories are reactivated in reverse order. This forward and backward rehearsal has been linked to processes such as planning, memory consolidation, and decision-making [46,47]. Altogether, it suggests that the minimalization of the time dimension might be a general mechanism for predictive representation in the brain.

Our findings on CIR in rats align with these neural processes, as they suggest that dynamic stimuli are processed in a manner that encodes predictions of future interactions. This predictive mechanism may correspond to forward replay in place-cell activity, where neural sequences pre-play trajectories in anticipation of upcoming interactions. Similarly, the ability of rats to generalize learned associations to novel stimuli may involve backward replay, as past spatial interactions are reactivated to inform current decision-making. By linking behavioral performance with these mechanisms, our study offers a bridge between CIR as a cognitive construct and hippocampal neural activity during forward and backward rehearsal.

In addition, hippocampal cells in freely flying bats encode their future positions strongly related to intersections between trajectories [31], as predicted by the time compaction hypothesis. In the Dotson and Yarstev study [31], the neurons fired concerning nonlocal subjects’ positions in the environment. They represented the future locations of the bats, both during random foraging and goal-directed navigation. Moreover, most of these cells had their firing fields located at the intersections of the flying paths. These intersections correspond to the CIRs proposed by the time compaction mechanism. Their overrepresentation within the hippocampus suggests their high importance for navigation, and the results obtained in our study indicate a similar role for the predicted collision locations of moving objects for representing dynamic visual environments.

In this study, we did not demonstrate that our task depends on the hippocampus. However, our previous research has shown that hippocampal inactivation impairs the discrimination of visual object positions using the same apparatus [12]. Additionally, our unpublished data indicate that hippocampal CA1 principal cells encode the positions of visual stimuli in the present VSD task, suggesting critical hippocampal involvement and making the task suitable for testing the time compaction hypothesis.

Our findings reveal that the rat’s brain can create abstract representations of inaccessible spaces where future interactions are spatially arranged. This is one of the central predictions of the time compaction hypothesis. Its experimental confirmation makes the existence of the compact internal representation as a cognitive mechanism to deal with dynamic situations plausible. In general, according to the time compaction hypothesis, when the subject is part of a dynamic situation (e.g., when it moves among other subjects), the CIR is a static map containing, besides the predicted interactions, the potential actions to deal with the situation (i.e., the pathways to be followed) [18]. In the same way, conventional cognitive maps mentally represent static environments by spatially mapping actual objects and trajectories to be navigated [44,48,49]. Thus, considering that the position of a static object will be the location of the future potential interactions of the agent with the object, the CIR would be a generalization of the cognitive map for time-changing scenarios [37]. We recognize that our results may be explained by predictions based on visuospatial memory associations rather than the presence of a fully formed static map. However, the presented behavioral evidence supports a single general mechanism capable of mentally representing dynamic and static situations. Evidence for CIR in humans has been established [20], and similar findings in rats suggest that shared cognitive mechanisms may exist across species, supporting CIR as a generalizable principle. Rodent models allow invasive techniques, like electrophysiology and optogenetics, to uncover neural mechanisms underlying CIR, offering deeper mechanistic insights, and our study opens a new venue to the study of spatiotemporal cognition, as the time compaction hypothesis claims that the same neural populations responsible for processing static spaces would be involved in processing dynamic situations by spatially representing future interactions.

Recent theories propose that cognitive maps do not represent only explicit maps of space but can predict possible future states, e.g., possible future rewards or spatial positions in the environment [50]. Positional information about future locations may be more critical for survival than current positions. Predictive cognitive maps could utilize successor representations [51]. Both rats and humans demonstrate analogous navigation choices and trajectory patterns, mirroring the behavior of reinforcement learning agents utilizing successor representation to adapt effectively to changing environments [52]. These predictive capabilities of the hippocampus and its central role in generating cognitive maps and processing static and dynamic situations make this area the prime candidate for characterizing the cell populations responsible for the CIR.

### Partial visual overlap of novel and familiar stimuli in the dynamic VSD task generalization is not the only factor influencing the discrimination of novel stimuli

In the generalization tests of the dynamic VSD task, the design of the familiar and novel stimuli raises a possible explanation of whether the rats’ generalization performance could be based purely on the direct transference of partial visuospatial information: the familiar Dynamic REW stimulus and the novel Compl. to REW stimulus share the circle moving vertically from top to bottom (Fig 3A). This common feature could eventually trigger the lever-pressing behavior when the Compl. to REW stimulus was presented, explaining the reported increased lever-pressing probability during it compared to other novel stimuli. However, this design allows us to compare our results to previous studies assessing the generalization of partially visually overlapping stimuli and eventually provide further supporting evidence for the time compaction mechanism in rats. The lever-pressing probability for the dynamic Compl. to REW stimulus during the generalization tests is higher than the probability for the Dynamic REW stimulus (Fig 3B and 3E) even though the rats were already used to dynamic stimuli from the training phase (in contrast to the generalization of the static VSD task) and the novelty effect is likely comparatively lower. However, their performance significantly drops when rats discriminate novel visual scenes in which part of the familiar rewarded stimulus is relocated [29], the stimulus is turned upside down [30], or when discriminating novel visual shapes, which were, in fact, partially rearranged familiar rewarded stimuli [22]. The novel stimuli in our generalization tests of the dynamic version of the VSD task are also partially rearranged or relocated familiar stimuli. Therefore, based on the previous findings, we would expect a lower probability of lever-pressing during the presentation of the Compl. to REW stimulus compared to the Dynamic REW stimulus if generalization relied solely on their shared visual features. However, the reverse was true, indicating the partial visual similarity between the Dynamic REW and Compl. to REW stimuli was not the only factor influencing generalization. The results suggest that the rats did not generalize the stimuli based solely on visual similarity. We propose that another mechanism, compatible with encoding future interactions hypothesized by the time compaction, is involved in discriminating novel dynamic stimuli in our VSD task.

### The novelty effect but not the initial configuration impacts the generalization of stimuli in the VSD task

Control 1 stimulus in the dynamic VSD task generalization had the starting positions of the moving circles identical to the locations of the familiar Static REW and Static non-REW stimulus, respectively. Therefore, the rats could perceive the Control 1 stimulus as potentially rewarded because the starting position of one circle was previously rewarded, although the starting position of the other circle was identical to the previously non-rewarded static position. However, the rats’ lever-pressing probability was lower during the presentation of the novel Compl. to non-REW stimulus, which shared the position of predicted collision with the Dynamic non-REW stimulus from the training phase, compared to both novel control stimuli (Control 1, Control 2) in the generalization tests of the dynamic VSD task (Fig 3E and 3F). In addition, there was no difference in the probability of lever-pressing between the two novel control stimuli (Fig 3E and 3F). Altogether, these results suggest that the objects’ starting positions in our task do not present the crucial information used to generalize novel stimuli, but rather, they are the predicted intersection of the displayed moving objects.

During individual presentations of the novel stimuli in the generalization tests of the dynamic VSD task, the lever-pressing probability was initially equivalent for all four novel stimuli but changed over their duration. It gradually increased for the Compl. to REW stimulus and progressively decreased for the Compl. to non-REW stimulus, respectively. At the same time, it stayed similar for the whole duration of both control stimuli (Fig 3F). The equal probability of lever-pressing for all four novel stimuli within the first few seconds of their presentations complies with the natural tendency of rats to respond to novel stimuli [53,54]. However, after the first two (1 s long) repetitions of circle approaches during novel stimuli, when the rats could estimate the predicted collision position more reliably, they clearly generalized the Compl. to REW and Compl. to non-REW stimuli, and we observed very similar results in the generalization tests of the static VSD task. These results support the previously mentioned interpretation that the position of the predicted collision is crucial for discriminating and generalizing the dynamic visuospatial stimuli in our task.

### Rats learn to discriminate dynamic visuospatial stimuli faster, with better accuracy and shorter reaction time than static visuospatial stimuli

Acting swiftly and precisely in complex dynamic surroundings is an essential ability. Natural ecosystems are constantly changing and responding especially to dynamic elements of the environment (e.g., an approaching predator, a falling boulder, etc.) is crucial. At the same time, static objects usually do not pose an immediate threat. Therefore, processing primarily dynamic events quickly and effectively would be beneficial from an ecological standpoint.

In the learning phase of the experiment, we found that the rats learned to discriminate the dynamic stimuli faster (reflected by the steeper learning curve slope), with better accuracy (reflected by the higher lever-pressing probability at the end of the first training configuration), and with shorter reaction times (reflected by the shorter time to the first lever press after stimulus onset) than the static ones. Our results agree with previous human studies reporting that participants detect moving objects more accurately than their static versions [55] and that drivers detect moving objects sooner than static objects [56]. In addition, greater attention was observed to moving objects than static objects in human infants [57], adult human subjects [58,59], and mice [60].

Abrams and Christ [58] proposed that the onset of movement, not the movement itself, captures attention. In our task, the dynamic stimuli consisted of 1 s long repetitions of individual approaches of two moving circles. Thus, the movement of the objects was repeatedly initiated during the dynamic stimuli presentations. This could attract even more attention to the dynamic stimuli and affect the rats’ performance in our task. However, we observed faster responses to the dynamic stimulus after its onset compared to the static stimulus only for the pair of rewarded stimuli but not for the pair of non-rewarded stimuli (Fig 4C). Nevertheless, given that the onset of movement is the critical period for identifying potential threats from moving objects, it may be essential to pay attention to it [58]. In another study, Smith and Abrams [61] conducted a series of experiments that confirmed attentional capturing by motion onset in humans and demonstrated its validity in two distinct scenarios: the animated movement of objects on a computer screen and the natural motion of real objects.

The higher complexity of dynamic stimuli in comparison to static stimuli in our VSD task (two objects vs. one object) was unlikely to contribute to faster learning as it was previously shown that rats learn to discriminate concurrent complex visual scenes with multiple objects with the same performance as concurrent single visual objects [62].

Altogether, these findings suggest that the ability to distinguish moving stimuli with superior performance, compared to static stimuli, could be a phenomenon shared among different species and an evolutionary advantage critical for survival in the ever-changing world.

## Conclusions

In summary, our results show the rats’ ability to generalize learned spatial associations to novel dynamic stimuli suggesting that rats can depict dynamic environments by creating static representations that capture significant expected interactions between objects. These insights advance our understanding of how animals process dynamic spatial information, though further research is needed to clarify the neural mechanisms driving these behaviors.

## Supporting information

S1 Appendix Supporting data analysis.
(DOCX)

S2 Appendix Asymptotic performance in the dynamic VSD task.
(DOCX)

S1 Fig Mean probability of pressing the lever within 1 s bins of the presentations of the four types of familiar stimuli during the 2nd-5th configuration (from left to right) of the training phase of the dynamic VSD task.
(A). Training stimuli for the VSD task - dynamic version: two complementary rewarded stimuli – one static and one dynamic (Static REW and Dynamic REW, in red rectangle) and two complementary non-rewarded stimuli (Static non-REW and Dynamic non-REW). (B) Points represent mean ± SEM. Lines represent predictions from logistic regression (GLMM with binomial distribution and logit link function with grouping variable the rat id. Fixed factors: stimulus type, normalized time, and their interaction).
(TIF)

S2 Fig Hazard Ratio (HR) for the four types of familiar stimuli during the 2nd-5th configuration (from left to right) of the training phase of the dynamic VSD task.
(A). Training stimuli for the VSD task - dynamic version. (B) Points represent HR ± SEM from Cox Proportional Hazards regression models at different training times (grouping variable the rat id and stimulus type as a fixed factor). Lines represent HR trends with normalized time from linear regression (only for illustrative purposes).
(TIF)

S3 Fig Median survival time (i.e., the median reaction time of pressing the lever for the first time after the stimulus onset) for the four types of familiar stimuli during the 2nd-5th configuration (from left to right) of the training phase of the dynamic VSD task.
(A). Training stimuli for the VSD task - dynamic version. (B) Points represent Median Survival Time ± SEM for each stimulus type from survival curves at different training times. Median survival time means the shortest survival time for which the survivor function is less than or equal to 0.5. Lines represent trends of Median Survival Time with normalized time from linear regression (only for illustrative purposes).
(TIF)

S4 Fig Equal discrimination of dynamic and static stimulus during the asymptotic performance of the dynamic VSD task.
(A) Rewarded (in red rectangle) and non-rewarded stimuli presented in the training sessions of the dynamic VSD task. (B) Mean probability of pressing the lever within 1 s bins of the presentations of individual stimuli during the last session of the final training configuration. (C) Lever-pressing probability distribution throughout individual stimuli duration (histograms binned at 200 ms and solid lines as in Fig 2). (D) Probability of the first lever press throughout individual stimuli duration after the stimulus onset during the last session of the final training configuration. The black hashtag marks significant differences (p < 0.0001) between rewarded and non-rewarded stimuli. Data in panels B and D are shown as means ± SEM.
(TIF)

S1 Table The design of the behavioral configurations used for training in the VSD task.
Configurations 1st – 5th were the training configurations, and Gen was the configuration for generalization tests. Time/stimulus shows the duration of a single stimulus in the individual configuration. In each session, two rewarded and two non-rewarded familiar stimuli were presented in the same number. Reinforcement: CR = continuous reinforcement, the reward was delivered after every correct lever press; FR2 = fixed ratio 2, the reward was delivered after every second correct lever press; VR3 = variable ratio 3, the reward was delivered after three presses on average but never after more than five correct presses. Session duration shows the total time to complete a single session. Pauses refer to the blank screen periods between individual stimuli presentations. For the generalization test, 3- and 5-s pauses were pseudorandomly presented between individual stimuli. S REW = the number of presentations of each type of rewarded stimulus during the session, S non-REW = the number of presentations of each type of non-rewarded stimulus during the session, and S GEN = the number of presentations of each novel stimulus during the generalization test. *novel stimuli in the generalization test were not rewarded.
(DOCX)

S2 Table The learning progress in the dynamic version of the VSD task.
The number of sessions to achieve stable performance in the respective configuration of the task and the total number of training sessions for each rat to reach the criteria in the final (5th) training configuration. Rat 40 was excluded from the training after 45 sessions as it could not reach a stable performance of > 60% in the first configuration of the task.
(DOCX)

S3 Table Comprehensive statistical data for all figures.
(DOCX)
