# Supplementary material for: Spatial prediction of dynamic interactions in rats
Source: PLoS One. 2025 Feb 25;20(2):e0319101. doi: 10.1371/journal.pone.0319101 (PMC11856586; doi:10.1371/journal.pone.0319101)
Supplement: S1 Appendix — (DOCX) [file pone.0319101.s001.docx]

**Supporting data analysis**

We calculated the mean probability as the average number of lever presses per second within the presentation periods. As this probability was not independent for each animal, data was analyzed using a generalized linear mixed model (GLMMN), taking the rat as a grouping factor (i.e., including a random intercept for the rat). Due to the dichotomous response variable (probability of pressing the lever), we used a binomial distribution with a logit link function (i.e., a logistic regression model). The stimulus type was the only fixed effect in analyzing the mean probability of lever presses during the asymptotic and generalization performance. The exponential value of each regression coefficient obtained from the model was the Odds Ratio (OR) of each stimulus type, i.e., the multiplying factor of the Odds of each stimulus type, which was then transformed into a probability. To study the learning dynamics of each type of stimulus, we also included the training time as a fixed factor and its interaction with the type of stimulus. As individual rats took different amounts of training sessions to reach the learning criteria during the first training configuration, this time was normalized between 0 and 1, with 0 being the first session and 1 being the last session in the first training configuration. Moreover, we included a random slope of time in the model. Time coefficients obtained from the models were interpreted in the same way. Their exponential values correspond to the OR (increase/decrease of the Odds during the entire training configuration) and were transformed into probability at each session/time.

Probability distribution was analyzed similarly, but in this case, the response variable was calculated as the number of lever presses per ms within 200 ms bins over each presentation period. A mixed effect logistic regression (GLMM with binomial distribution and logit link function) was also used, with the stimulus type, the time from the stimulus onset (binned into 200 ms periods), and its square as fixed factors, both in interaction with the stimulus type and with a random intercept with each rat and session as grouping factors. This analysis was only done to study asymptotic and generalization performance.

We calculated the time from the onset of the stimulus to the first lever press for each presentation period to analyze the reaction time. This kind of response variable (time-to-event) is suitably analyzed with Survival models. In this case, we chose a Cox Proportional Hazards model. The exponential of the coefficients obtained from the model corresponds to the Hazard Ratio (HR). The model calculates an empirical baseline hazard function over the time course of the presentation period (which corresponds to the hazard, i.e., the “risk” or probability of suffering the event, when all independent variables are 0) and assumes that a factor proportionally modifies this hazard over all time points of the presentation period. This way, at any time from the onset of the stimulus, the probability of pressing the lever (the risk in our case) is multiplied by the HR, so HRs greater than 1 implies a greater risk at any time, which in turn implies that it is more feasible that the rat presses the lever for the first time earlier, while HRs lower than 1 implies a decrease of the risk, so rats would tend to press the lever later. For analyses of asymptotic and generalization performance, we included the stimulus type as a fixed effect factor, and to avoid the lack of independence within rats, a random intercept was also included (taking the rat as a grouping factor). Data were plotted as cumulative hazard functions representing the probability that the rat has already pressed the lever for the first time (y-axis) at a certain time from the onset of the stimulus (x-axis). The faster the curve grows, the greater the HR, and the earlier the rat can press the lever for the first time. Median lifetime was also obtained, representing the median reaction time of the rats. A normalized time (as in above) was included as a fixed factor to get the HR for each stimulus type at each session of the training configuration to analyze learning dynamics. The HR of each stimulus type was referred to as its baseline risk, i.e., the risk of each stimulus type in the first session, so the probability or risk that appears in the y-axis is an arbitrary value (as risk is the function of the reaction time). Information about the dynamics is extracted from the curves’ shapes, so curves that grow faster imply that the reaction time decreases faster than those that grow slower.
