# Supplementary material for: Spatial prediction of dynamic interactions in rats
Source: PLoS One. 2025 Feb 25;20(2):e0319101. doi: 10.1371/journal.pone.0319101 (PMC11856586; doi:10.1371/journal.pone.0319101)
Supplement: S2 Appendix — (DOCX) [file pone.0319101.s002.docx]

**Asymptotic performance: equal discrimination performance for dynamic and static stimuli**

In the last session of the final training configuration, when the rats had already reached the asymptotic performance in the dynamic VSD task (see S1-S3 Figs for more details about the rats’ performance during training configurations 2-5), the lever-pressing probability during the rewarded stimuli was higher than during the non-rewarded stimuli (p < 0.0001 in all cases). However, there was no difference between the two rewarded (Dynamic REW vs. Static REW, p = 0.63) or non-rewarded stimuli (Dynamic non-REW vs. Static non-REW, p = 0.88) (S4B and S4C Figs).

Visual stimulus type affected the time to the first lever press after the stimulus onset (p < 0.0001; S4D Fig). Tukey posthoc tests showed a shorter time to the first operant response for the rewarded stimuli than for the non-rewarded stimuli (all p < 0.0001). Again, there was no difference between the two rewarded (p = 0.83) or non-rewarded stimuli (p = 1). The median time to the first lever press after the stimulus onset was 1760 ms and 2040 ms for the Dynamic REW and Static REW stimulus, respectively. The median time was longer than the stimulus duration for both non-rewarded stimuli.

The lack of differences in the lever-pressing probability and the median time to the first lever press after the stimulus onset between Dynamic REW and Static REW suggests that after extensive training, the rats can discriminate complementary dynamic and static visuospatial stimuli equally.
