# Supplementary figures and images for: Spatial prediction of dynamic interactions in rats
Source: PLoS One. 2025 Feb 25;20(2):e0319101. doi: 10.1371/journal.pone.0319101 (PMC11856586; doi:10.1371/journal.pone.0319101)

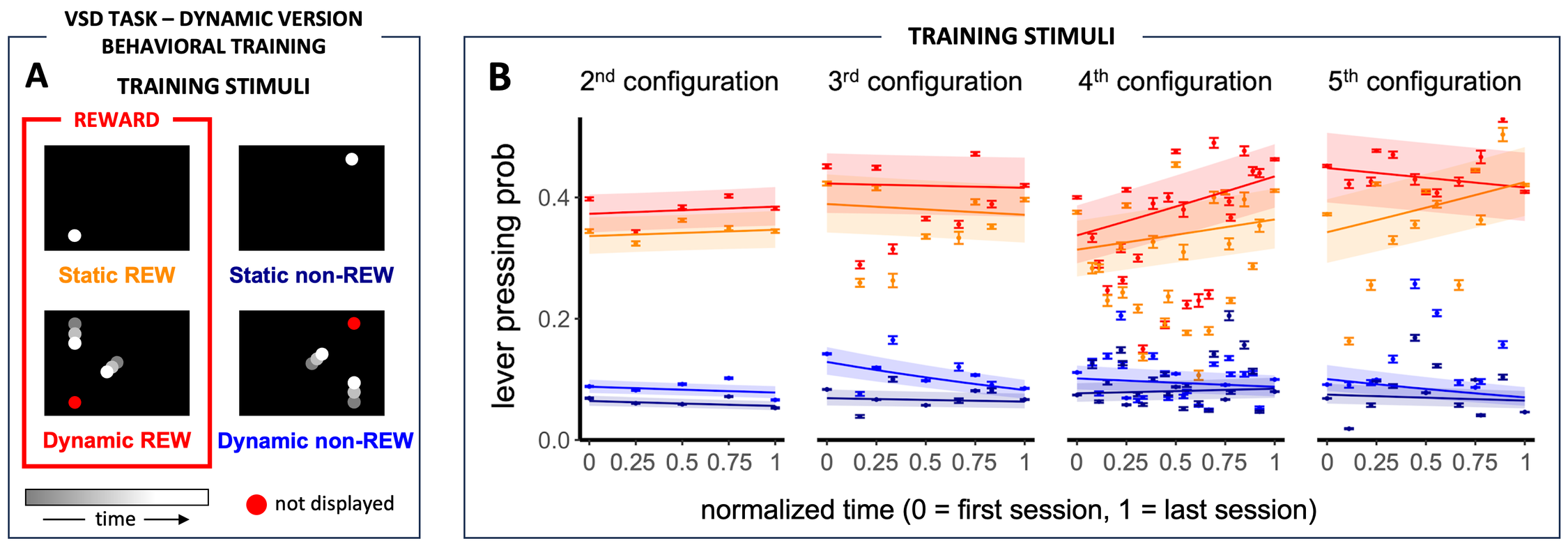

Supplement: S1 Fig — (A). Training stimuli for the VSD task - dynamic version: two complementary rewarded stimuli – one static and one dynamic (Static REW and Dynamic REW, in red rectangle) and two complementary non-rewarded stimuli (Static non-REW and Dynamic non-REW). (B) Points represent mean ± SEM. Lines represent predictions from logistic regression (GLMM with binomial distribution and logit link function with grouping variable the rat id. Fixed factors: stimulus type, normalized time, and their interaction). (TIF) [file pone.0319101.s003.tif]

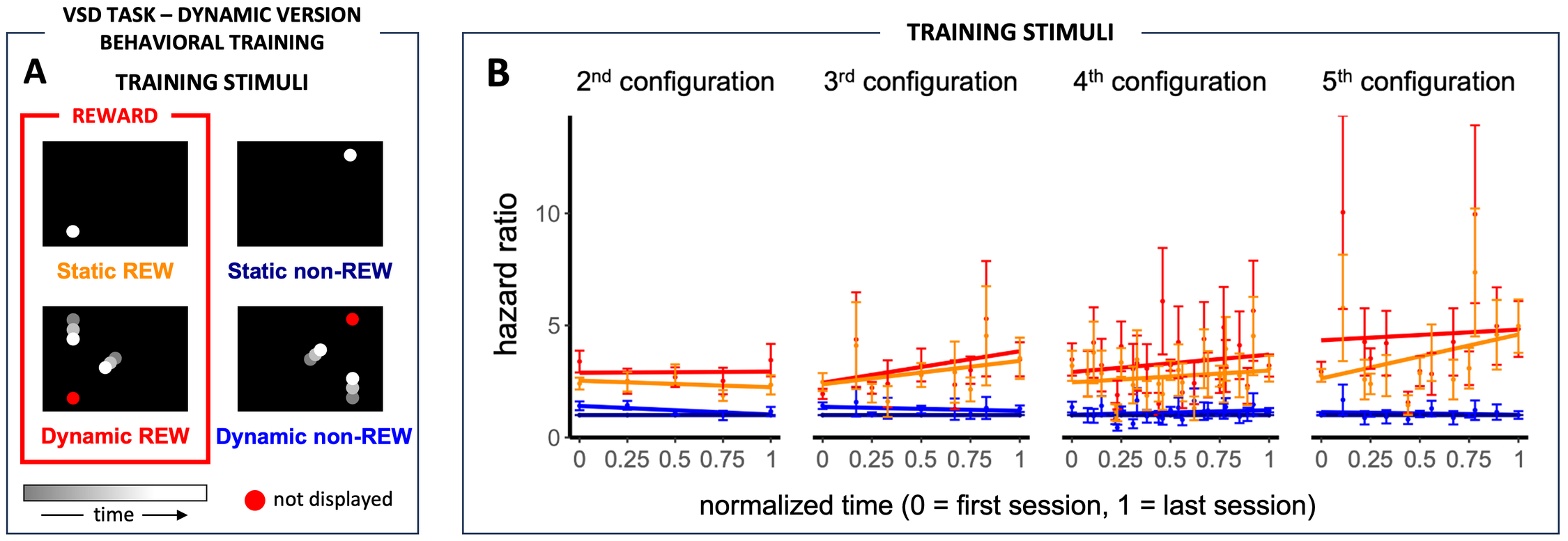

Supplement: S2 Fig — (A). Training stimuli for the VSD task - dynamic version. (B) Points represent HR ± SEM from Cox Proportional Hazards regression models at different training times (grouping variable the rat id and stimulus type as a fixed factor). Lines represent HR trends with normalized time from linear regression (only for illustrative purposes). (TIF) [file pone.0319101.s004.tif]

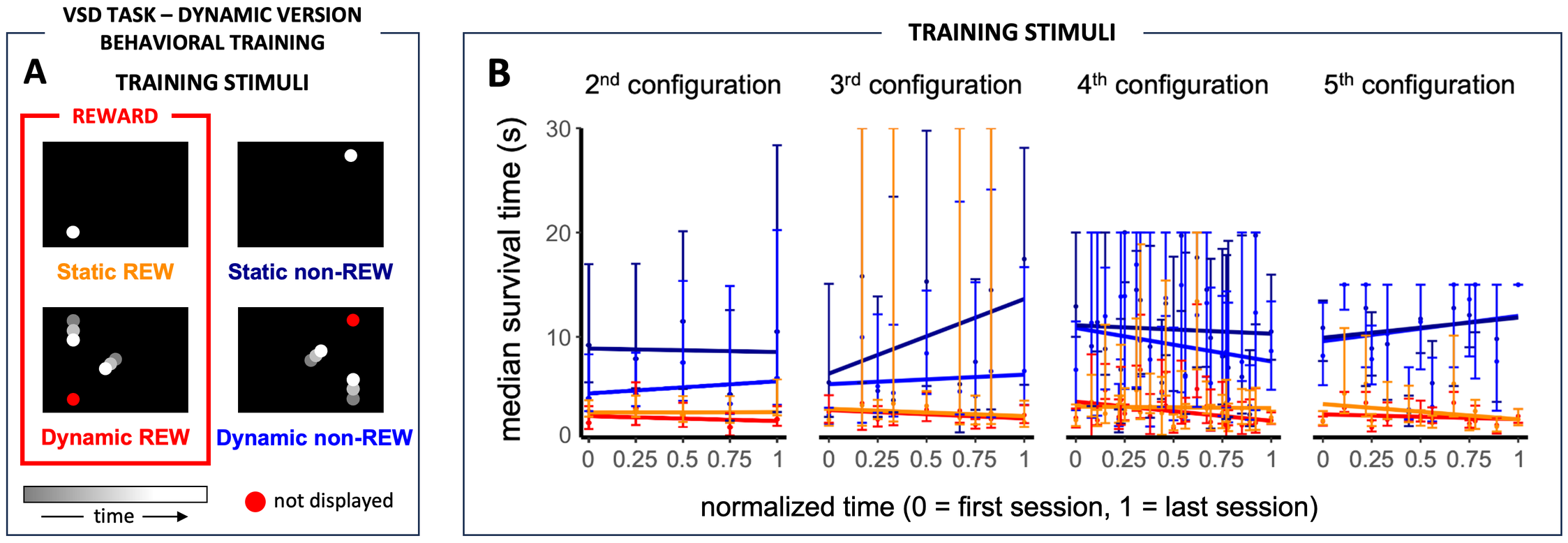

Supplement: S3 Fig — (A). Training stimuli for the VSD task - dynamic version. (B) Points represent Median Survival Time ± SEM for each stimulus type from survival curves at different training times. Median survival time means the shortest survival time for which the survivor function is less than or equal to 0.5. Lines represent trends of Median Survival Time with normalized time from linear regression (only for illustrative purposes). (TIF) [file pone.0319101.s005.tif]

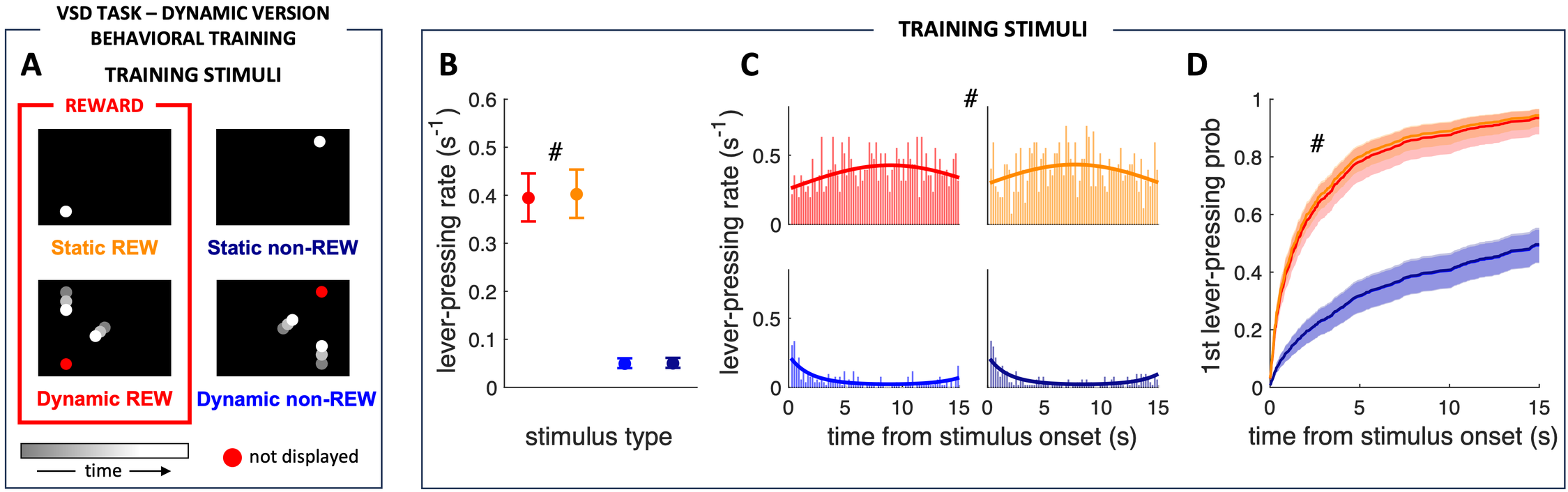

Supplement: S4 Fig — (A) Rewarded (in red rectangle) and non-rewarded stimuli presented in the training sessions of the dynamic VSD task. (B) Mean probability of pressing the lever within 1 s bins of the presentations of individual stimuli during the last session of the final training configuration. (C) Lever-pressing probability distribution throughout individual stimuli duration (histograms binned at 200 ms and solid lines as in Fig 2). (D) Probability of the first lever press throughout individual stimuli duration after the stimulus onset during the last session of the final training configuration. The black hashtag marks significant differences (p < 0.0001) between rewarded and non-rewarded stimuli. Data in panels B and D are shown as means ± SEM. (TIF) [file pone.0319101.s006.tif]
