# Supplementary material for: Spatial prediction of dynamic interactions in rats
Source: PLoS One. 2025 Feb 25;20(2):e0319101. doi: 10.1371/journal.pone.0319101 (PMC11856586; doi:10.1371/journal.pone.0319101)
Supplement: S1 Table — Configurations 1st – 5th were the training configurations, and Gen was the configuration for generalization tests. Time/stimulus shows the duration of a single stimulus in the individual configuration. In each session, two rewarded and two non-rewarded familiar stimuli were presented in the same number. Reinforcement: CR = continuous reinforcement, the reward was delivered after every correct lever press; FR2 = fixed ratio 2, the reward was delivered after every second correct lever press; VR3 = variable ratio 3, the reward was delivered after three presses on average but never after more than five correct presses. Session duration shows the total time to complete a single session. Pauses refer to the blank screen periods between individual stimuli presentations. For the generalization test, 3- and 5-s pauses were pseudorandomly presented between individual stimuli. S REW = the number of presentations of each type of rewarded stimulus during the session, S non-REW = the number of presentations of each type of non-rewarded stimulus during the session, and S GEN = the number of presentations of each novel stimulus during the generalization test. *novel stimuli in the generalization test were not rewarded. (DOCX) [file pone.0319101.s007.docx]

| Configuration | Time/Stimulus | Reinforcement | Session Duration | Pause | S REW | S non-REW | S GEN |
| --- | --- | --- | --- | --- | --- | --- | --- |
| 1st | 90 s | CR | 28 min 30 s | 5 s | 3 | 6 | --- |
| 2nd | 45 s | FR2 | 30 min | 5 s | 6 | 12 | --- |
| 3rd | 30 s | VR3 | 29 min 42 s | 3 s | 9 | 18 | --- |
| 4th | 20 s | VR3 | 34 min 30 s | 3 s | 15 | 30 | --- |
| 5th | 15 s | VR3 | 32 min 24 s | 3 s | 18 | 36 | --- |
| Gen | 15 + 10 s | VR3* | 45 min 24 s | 3 + 5 s | 18 | 36 | 12 |
