# Supplementary material for: Spatial prediction of dynamic interactions in rats
Source: PLoS One. 2025 Feb 25;20(2):e0319101. doi: 10.1371/journal.pone.0319101 (PMC11856586; doi:10.1371/journal.pone.0319101)
Supplement: S2 Table — The number of sessions to achieve stable performance in the respective configuration of the task and the total number of training sessions for each rat to reach the criteria in the final (5th) training configuration. Rat 40 was excluded from the training after 45 sessions as it could not reach a stable performance of > 60% in the first configuration of the task. (DOCX) [file pone.0319101.s008.docx]

| Animal | 1st config. | 2nd config. | 3rd config. | 4th config. | 5th config. | Total sessions |
| --- | --- | --- | --- | --- | --- | --- |
| rat 39 | 17 | 5 | 5 | 10 | 10 | 47 |
| rat 41 | 22 | 5 | 7 | 5 | 5 | 44 |
| rat 42 | 11 | 5 | 5 | 14 | 5 | 40 |
| rat 43 | 31 | 5 | 5 | 6 | 11 | 58 |
| rat 44 | 30 | 5 | 5 | 5 | 5 | 50 |
| rat 45 | 22 | 5 | 5 | 5 | 5 | 42 |
| rat 46 | 21 | 5 | 5 | 5 | 5 | 41 |
