# Supplementary material for: Spatial prediction of dynamic interactions in rats
Source: PLoS One. 2025 Feb 25;20(2):e0319101. doi: 10.1371/journal.pone.0319101 (PMC11856586; doi:10.1371/journal.pone.0319101)
Supplement: S3 Table — (DOCX) [file pone.0319101.s009.docx]

Fig 2B

|  | **lever-pressing prob.** | | | |
| --- | --- | --- | --- | --- |
| *Predictors* | *Odds Ratios* | *CI* | *Statistic* | *p* |
| Intercept | 0.05 | 0.04 – 0.07 | -26.07 | **7.056e-150** |
| Static REW | 6.53 | 6.10 – 6.98 | 54.69 | **0.000e+00** |
| **Random Effects** | | | | |
| σ^2^ | 3.29 | | | |
| τ_00_ _Rat_ | 0.07 | | | |
| ICC | 0.02 | | | |
| N _Rat_ | 6 | | | |
| Observations | 1296 | | | |
| Marginal R^2^ / Conditional R^2^ | 0.189 / 0.206 | | | |

Fig 2C

|  | **lever-pressing prob.** | | | |
| --- | --- | --- | --- | --- |
| *Predictors* | *Odds Ratios* | *CI* | *Statistic* | *p* |
| Intercept | 0.0000 | 0.0000 – 0.0000 | -118.6126 | **0.000e+00** |
| Static REW | 5.3336 | 4.9919 – 5.6988 | 49.5485 | **0.000e+00** |
| time (linear) | 0.0000 | 0.0000 – 0.0000 | -10.6510 | **1.725e-26** |
| time (quadratic) | 15447149.7698 | 773111.0030 – 308641883.3486 | 10.8333 | **2.393e-27** |
| time (linear) * Static REW | 33.3874 | 0.9185 – 1213.6893 | 1.9136 | 5.568e-02 |
| time (quadratic) * Static REW | 0.0000 | 0.0000 – 0.0000 | -10.3283 | **5.246e-25** |
| **Random Effects** | | | | |
| σ^2^ | 3.29 | | | |
| τ_00_ _Rat_ | 0.05 | | | |
| ICC | 0.01 | | | |
| N _Rat_ | 6 | | | |
| Observations | 3600 | | | |
| Marginal R^2^ / Conditional R^2^ | 0.193 / 0.204 | | | |

Figs 2D and 2G

=====================================================================

| *contrast* | *estimate* | *SE* | *df* | *z.ratio* | *p* |
| --- | --- | --- | --- | --- | --- |
| Static non-REW vs. Static REW | -1.3580334 | 0.12827283 | Inf | -10.587070 | **3.674838e-14** |
| Compl. to non-REW vs. Compl. to REW | 0.1423708 | 0.08114048 | Inf | 1.754621 | 2.955109e-01 |

Fig 2E

|  | **lever-pressing prob.** | | | |
| --- | --- | --- | --- | --- |
| *Predictors* | *Odds Ratios* | *CI* | *Statistic* | *p* |
| Intercept | 0.28 | 0.18 – 0.43 | -5.61 | **2.035e-08** |
| Compl. to REW | 1.24 | 1.13 – 1.35 | 4.80 | **1.598e-06** |
| **Random Effects** | | | | |
| σ^2^ | 3.29 | | | |
| τ_00_ _Rat_ | 0.31 | | | |
| ICC | 0.09 | | | |
| N _Rat_ | 6 | | | |
| Observations | 576 | | | |
| Marginal R^2^ / Conditional R^2^ | 0.003 / 0.089 | | | |

Fig 2F

|  | **lever-pressing prob.** | | | |
| --- | --- | --- | --- | --- |
| *Predictors* | *Odds Ratios* | *CI* | *Statistic* | *p* |
| Intercept | 0.0001 | 0.0001 – 0.0001 | -55.8065 | **0.000e+00** |
| Compl. to REW | 1.1961 | 1.1097 – 1.2893 | 4.6790 | **2.883e-06** |
| time (linear) | 0.0000 | 0.0000 – 0.0000 | -9.1829 | **4.195e-20** |
| time (quadratic) | 0.5374 | 0.0366 – 7.8839 | -0.4532 | 6.504e-01 |
| time (linear) * Compl. to REW | 6554.9091 | 154.4497 – 278193.0936 | 4.5954 | **4.319e-06** |
| time (quadratic) * Compl. to REW | 0.0385 | 0.0010 – 1.5163 | -1.7378 | 8.224e-02 |
| **Random Effects** | | | | |
| σ^2^ | 3.29 | | | |
| τ_00_ _Rat_ | 0.17 | | | |
| ICC | 0.05 | | | |
| N _Rat_ | 6 | | | |
| Observations | 2400 | | | |
| Marginal R^2^ / Conditional R^2^ | 0.014 / 0.061 | | | |

Fig 3B

|  | lever-pressing prob. | | | |
| --- | --- | --- | --- | --- |
| Predictors | Odds Ratios | CI | Statistic | p |
| Intercept | 0.05 | 0.04 – 0.08 | -14.57 | 4.582e-48 |
| Static non-REW | 1.00 | 0.86 – 1.17 | 0.04 | 9.695e-01 |
| Dynamic REW | 16.70 | 14.68 – 19.00 | 42.72 | 0.000e+00 |
| Static REW | 12.20 | 10.72 – 13.89 | 37.87 | 0.000e+00 |
| Random Effects | | | | |
| σ2 | 3.29 | | | |
| τ00 session:Rat | 0.05 | | | |
| τ00 Rat | 0.20 | | | |
| ICC | 0.07 | | | |
| N session | 2 | | | |
| N Rat | 6 | | | |
| Observations | 48 | | | |
| Marginal R2 / Conditional R2 | 0.339 / 0.385 | | | |

Pairwise comparisons

| contrast | odds.ratio | SE | df | null | z.ratio | p |
| --- | --- | --- | --- | --- | --- | --- |
| Dynamic non-REW vs. Static non-REW | 0.99707804 | 0.076214432 | Inf | 1 | -0.03828262 | 9.694623e-01 |
| Dynamic non-REW vs. Dynamic REW | 0.05987785 | 0.003946266 | Inf | 1 | -42.71963201 | 0.000000e+00 |
| Dynamic non-REW vs. Static REW | 0.08194333 | 0.005413820 | Inf | 1 | -37.86602802 | 0.000000e+00 |
| Static non-REW vs. Dynamic REW | 0.06005332 | 0.003954444 | Inf | 1 | -42.71177528 | 0.000000e+00 |
| Static non-REW vs. Static REW | 0.08218347 | 0.005425071 | Inf | 1 | -37.85391029 | 0.000000e+00 |
| Dynamic REW vs. Static REW | 1.36850819 | 0.070865494 | Inf | 1 | 6.05837982 | 1.374994e-09 |

Fig 3C

|  | lever-pressing prob. | | | |
| --- | --- | --- | --- | --- |
| Predictors | Odds Ratios | CI | Statistic | p |
| Intercept | 0.00 | 0.00 – 0.00 | -88.06 | 0.000e+00 |
| Static non-REW | 1.02 | 0.87 – 1.21 | 0.29 | 7.695e-01 |
| Dynamic REW | 9.84 | 8.66 – 11.19 | 34.95 | 1.244e-267 |
| Static REW | 8.31 | 7.30 – 9.47 | 31.92 | 1.366e-223 |
| time (linear) | 0.00 | 0.00 – 0.01 | -3.89 | 1.002e-04 |
| time (quadratic) | 23339525181009.96 | 60331972374.91 – 9028934646624608.00 | 10.13 | 4.245e-24 |
| time (linear) * Static non-REW | 1.40 | 0.00 – 2379.91 | 0.09 | 9.289e-01 |
| time (linear) * Dynamic REW | 117912.78 | 272.35 – 51049481.30 | 3.77 | 1.631e-04 |
| time (linear) * Static REW | 87937.96 | 165.49 – 46727826.73 | 3.56 | 3.771e-04 |
| time (quadratic) * Static non-REW | 0.12 | 0.00 – 471.24 | -0.51 | 6.108e-01 |
| time (quadratic) * Dynamic REW | 0.00 | 0.00 – 0.00 | -9.87 | 5.792e-23 |
| time (quadratic) * Static REW | 0.00 | 0.00 – 0.00 | -10.25 | 1.231e-24 |
| Random Effects | | | | |
| σ2 | 3.29 | | | |
| τ00 session:Rat | 0.02 | | | |
| τ00 Rat | 0.07 | | | |
| ICC | 0.03 | | | |
| N session | 2 | | | |
| N Rat | 6 | | | |
| Observations | 3600 | | | |
| Marginal R2 / Conditional R2 | 0.284 / 0.304 | | | |

Pairwise comparisons

| contrast | term | OR | SE | z.ratio | p |
| --- | --- | --- | --- | --- | --- |
| Dynamic non-REW vs. Static non-REW | 1 | 0.99999866 | 1.459440e-05 | -0.09148001 | 9.271112e-01 |
| Dynamic non-REW vs. Dynamic REW | 1 | 0.99995455 | 1.191572e-05 | -3.81417909 | 1.366364e-04 |
| Dynamic non-REW vs. Static REW | 1 | 0.99995565 | 1.231845e-05 | -3.60034419 | 3.177962e-04 |
| Static non-REW vs. Dynamic REW | 1 | 0.99995589 | 1.189322e-05 | -3.70913943 | 2.079649e-04 |
| Static non-REW vs. Static REW | 1 | 0.99995699 | 1.224196e-05 | -3.51377893 | 4.417803e-04 |
| Dynamic REW vs. Static REW | 1 | 1.00000110 | 9.013458e-06 | 0.12182238 | 9.030397e-01 |
| Dynamic non-REW vs. Static non-REW | 2 | 1.00000000 | 4.217872e-09 | 0.50900046 | 6.107519e-01 |
| Dynamic non-REW vs. Dynamic REW | 2 | 1.00000003 | 3.404810e-09 | 9.86691942 | 5.791599e-23 |
| Dynamic non-REW vs. Static REW | 2 | 1.00000004 | 3.483361e-09 | 10.24619502 | 1.230942e-24 |
| Static non-REW vs. Dynamic REW | 2 | 1.00000003 | 3.318415e-09 | 9.47683993 | 2.621002e-21 |
| Static non-REW vs. Static REW | 2 | 1.00000003 | 3.392009e-09 | 9.88921286 | 4.636591e-23 |
| Dynamic REW vs. Static REW | 2 | 1.00000000 | 2.309866e-09 | 0.90750264 | 3.641411e-01 |

Fig 3D

|  | lever-pressing prob. | | | |
| --- | --- | --- | --- | --- |
| Predictors | Estimates | CI | Statistic | p |
| Static non-REW | 0.99 | 0.82 – 1.21 | -0.08 | 9.340e-01 |
| Dynamic REW | 5.84 | 4.75 – 7.17 | 12.67 | 0.000e+00 |
| Static REW | 4.47 | 3.65 – 5.47 | 10.40 | 0.000e+00 |
| Observations | 1296 | | | |
| R2 Nagelkerke | 0.289 | | | |

Pairwise comparisons

| contrast | ratio | SE | df | null | z.ratio | p |
| --- | --- | --- | --- | --- | --- | --- |
| Dynamic non-REW vs. Static non-REW | 1.0066244 | 0.08019905 | Inf | 1 | 0.08287201 | 9.339533e-01 |
| Dynamic non-REW vs. Dynamic REW | 0.1713028 | 0.02385661 | Inf | 1 | -12.66874708 | 8.811047e-37 |
| Dynamic non-REW vs. Static REW | 0.2237494 | 0.03220027 | Inf | 1 | -10.40376497 | 2.383264e-25 |
| Static non-REW vs. Dynamic REW | 0.1701755 | 0.02379838 | Inf | 1 | -12.66338595 | 9.434081e-37 |
| Static non-REW vs. Static REW | 0.2222770 | 0.03153084 | Inf | 1 | -10.60127147 | 2.939562e-26 |
| Dynamic REW vs. Static REW | 1.3061633 | 0.08476208 | Inf | 1 | 4.11585539 | 3.857461e-05 |

Fig 3E

|  | lever-pressing prob. | | | |
| --- | --- | --- | --- | --- |
| Predictors | Odds Ratios | CI | Statistic | p |
| Intercept | 0.54 | 0.33 – 0.91 | -2.33 | 1.978e-02 |
| Compl. to non-REW | 0.74 | 0.63 – 0.87 | -3.61 | 3.090e-04 |
| Compl. to REW | 1.95 | 1.67 – 2.28 | 8.40 | 4.309e-17 |
| Control 2 | 0.89 | 0.76 – 1.04 | -1.43 | 1.514e-01 |
| Random Effects | | | | |
| σ2 | 3.29 | | | |
| τ00 Rat | 0.39 | | | |
| ICC | 0.11 | | | |
| N Rat | 6 | | | |
| Observations | 48 | | | |
| Marginal R2 / Conditional R2 | 0.036 / 0.138 | | | |

Pairwise comparisons

| contrast | odds.ratio | SE | df | null | z.ratio | p |
| --- | --- | --- | --- | --- | --- | --- |
| Control 1 vs. Compl. to non-REW | 1.3505941 | 0.11251571 | Inf | 1 | 3.607618 | 3.090207e-04 |
| Compl. to REW vs. Control 1 | 1.9539379 | 0.04079155 | Inf | 1 | -8.404164 | 4.309188e-17 |
| Control 1 vs. Control 2 | 1.1248227 | 0.09223431 | Inf | 1 | 1.434474 | 1.514371e-01 |
| Compl. to REW vs. Compl. to non-REW | 2.6389771 | 0.03103140 | Inf | 1 | -11.849770 | 2.157822e-32 |
| Compl. to non-REW vs. Control 2 | 0.8328355 | 0.06990788 | Inf | 1 | -2.179176 | 2.931858e-02 |
| Compl. to REW vs. Control 2 | 2.1978334 | 0.17683582 | Inf | 1 | 9.787228 | 1.277503e-22 |

Fig 3F

|  | lever-pressing prob. | | | |
| --- | --- | --- | --- | --- |
| Predictors | Odds Ratios | CI | Statistic | p |
| Intercept | 0.00 | 0.00 – 0.00 | -66.73 | 0.000e+00 |
| Compl. to non-REW | 0.81 | 0.72 – 0.93 | -3.11 | 1.846e-03 |
| Compl. to REW | 1.40 | 1.25 – 1.56 | 5.75 | 8.782e-09 |
| Control 2 | 0.93 | 0.82 – 1.05 | -1.12 | 2.632e-01 |
| time (linear) | 5.16 | 0.06 – 428.91 | 0.73 | 4.669e-01 |
| time (quadratic) | 0.10 | 0.00 – 10.34 | -0.97 | 3.337e-01 |
| time (linear) * Compl. to non-REW | 0.00 | 0.00 – 0.04 | -2.95 | 3.148e-03 |
| time (linear) * Compl. to REW | 4631.22 | 13.37 – 1604672.74 | 2.83 | 4.670e-03 |
| time (linear) * Control 2 | 0.52 | 0.00 – 293.78 | -0.20 | 8.379e-01 |
| time (quadratic) * Compl. to non-REW | 29.70 | 0.03 – 26380.00 | 0.98 | 3.276e-01 |
| time (quadratic) * Compl. to REW | 0.05 | 0.00 – 22.55 | -0.95 | 3.446e-01 |
| time (quadratic) * Control 2 | 1.72 | 0.00 – 1281.54 | 0.16 | 8.716e-01 |
| Random Effects | | | | |
| σ2 | 3.29 | | | |
| τ00 Rat | 0.11 | | | |
| ICC | 0.03 | | | |
| N Rat | 6 | | | |
| Observations | 2401 | | | |
| Marginal R2 / Conditional R2 | 0.018 / 0.050 | | | |

Pairwise comparisons

| contrast | degree | estimate | SE | df | z.ratio | p |
| --- | --- | --- | --- | --- | --- | --- |
| Control 1 vs. Compl. to non-REW | linear | 1.0000707 | 2.349510e-05 | Inf | 3.0086850 | 2.623810e-03 |
| Control 1 vs. Compl. to REW | linear | 0.9999400 | 2.095021e-05 | Inf | -2.8645382 | 4.176177e-03 |
| Control 1 vs. Control 2 | linear | 1.0000048 | 2.266333e-05 | Inf | 0.2112278 | 8.327095e-01 |
| Compl. to non-REW vs. Compl. to REW | linear | 0.9998693 | 2.214928e-05 | Inf | -5.9009601 | 3.613923e-09 |
| Compl. to non-REW vs. Control 2 | linear | 0.9999341 | 2.376889e-05 | Inf | -2.7726257 | 5.560604e-03 |
| Compl. to REW vs. Control 2 | linear | 1.0000648 | 2.120489e-05 | Inf | 3.0558894 | 2.243940e-03 |
| Control 1 vs. Compl. to non-REW | quadratic | 1.0000000 | 9.046145e-09 | Inf | -0.9789955 | 3.275822e-01 |
| Control 1 vs. Compl. to REW | quadratic | 1.0000000 | 8.017694e-09 | Inf | 0.9450805 | 3.446178e-01 |
| Control 1 vs. Control 2 | quadratic | 1.0000000 | 8.808230e-09 | Inf | -0.1616412 | 8.715884e-01 |
| Compl. to non-REW vs. Compl. to REW | quadratic | 1.0000000 | 8.407076e-09 | Inf | 1.9547226 | 5.061582e-02 |
| Compl. to non-REW vs. Control 2 | quadratic | 1.0000000 | 9.134508e-09 | Inf | 0.8136577 | 4.158411e-01 |
| Compl. to REW vs. Control 2 | quadratic | 1.0000000 | 8.142118e-09 | Inf | -1.1055033 | 2.689416e-01 |

Fig 3G

|  | lever-pressing prob. | | | |
| --- | --- | --- | --- | --- |
| Predictors | Estimates | CI | Statistic | p |
| Compl. to non-REW | 1.11 | 0.86 – 1.42 | 0.77 | 4.392e-01 |
| Compl. to REW | 1.15 | 0.90 – 1.48 | 1.64 | 1.020e-01 |
| Control 2 | 0.97 | 0.76 – 1.25 | -0.26 | 7.978e-01 |
| Observations | 575 | | | |
| R2 Nagelkerke | 0.004 | | | |

Analysis of Deviance Table (Type II tests)

Df Chisq Pr(>Chisq)

stimuli 3 4.4878 0.2134

Pairwise comparisons

| contrast | ratio | SE | df | null | z.ratio | p |
| --- | --- | --- | --- | --- | --- | --- |
| Control 1 vs. Compl. to non-REW | 0.9020726 | 0.12019431 | Inf | 1 | -0.7734800 | 0.43923837 |
| Control 1 vs. Compl. to REW | 0.8667990 | 0.07577366 | Inf | 1 | -1.6352290 | 0.10200105 |
| Control 1 vs. Control 2 | 1.0291415 | 0.11538432 | Inf | 1 | 0.2562051 | 0.79779246 |
| Compl. to non-REW vs. Compl. to REW | 0.9608973 | 0.11548069 | Inf | 1 | -0.3319002 | 0.73996463 |
| Compl. to non-REW vs. Control 2 | 1.1408633 | 0.16323087 | Inf | 1 | 0.9210820 | 0.35700763 |
| Compl. to REW vs. Control 2 | 1.1872896 | 0.11708378 | Inf | 1 | 1.7408531 | 0.08170933 |

Fig 4B

|  | **lever-pressing prob.** | | | |
| --- | --- | --- | --- | --- |
| *Predictors* | *Odds Ratios* | *CI* | *Statistic* | *p* |
| Intercept | 0.1750 | 0.1577 – 0.1942 | -32.7626 | **2.009e-235** |
| Static REW | 0.9988 | 0.9336 – 1.0737 | 0.0337 | 9.731e-01 |
| time | 2.6818 | 2.2491 – 3.1979 | 10.9868 | **4.421e-28** |
| time * Static REW | 1,1761 | 0.7591 – 0.9524 | -2.8023 | **5.075e-03** |
| **Random Effects** | | | | |
| σ^2^ | 3.29 | | | |
| τ_00_ _ID_ | 0.09 | | | |
| ICC | 0.03 | | | |
| N _ID_ | 154 | | | |
| Observations | 924 | | | |
| Marginal R^2^ / Conditional R^2^ | 0.022 / 0.048 | | | |

|  | **lever-pressing prob.** | | | |
| --- | --- | --- | --- | --- |
| *Predictors* | *Odds Ratios* | *CI* | *Statistic* | *p* |
| Intercept | 0.2088 | 0.1836 – 0.2376 | -23.8203 | **2.057e-125** |
| Static non-REW | 0.8099 | 0.7667 – 0.8556 | -7.5265 | **5.212e-14** |
| time | 0.4391 | 0.3515 – 0.5486 | -7.2451 | **4.322e-13** |
| time * Static non-REW | 1.0549 | 0.9520 – 1.1688 | 1.0207 | 3.074e-01 |
| **Random Effects** | | | | |
| σ^2^ | 3.29 | | | |
| τ_00_ _ID_ | 0.16 | | | |
| ICC | 0.05 | | | |
| N _ID_ | 154 | | | |
| Observations | 1848 | | | |
| Marginal R^2^ / Conditional R^2^ | 0.019 / 0.065 | | | |

Fig 4C

========================================================================

| *contrast* | *estimate* | *SE* | *df* | *z.ratio* | *p* |
| --- | --- | --- | --- | --- | --- |
| Static non-REW vs. Dynamic non-REW | 0.9656193 | 0.07453519 | Inf | -0.4693837 | 6.387954e-01 |
| Static non-REW vs. Dynamic REW | 0.3067653 | 0.19329424 | Inf | -6.1133336 | **9.757110e-10** |
| Static non-REW vs. Static REW | 0.3957896 | 0.20918673 | Inf | -4.4308380 | **9.386758e-06** |
| Dynamic non-REW vs. Dynamic REW | 0.3176877 | 0.17521448 | Inf | -6.5444738 | **5.970522e-11** |
| Dynamic non-REW vs. Static REW | 0.4098816 | 0.19207902 | Inf | -4.6433333 | **3.428326e-06** |
| Dynamic REW vs. Static REW | 1.2902031 | 0.11660587 | Inf | 2.1851356 | **2.887891e-02** |

S4B Fig

|  | **lever-pressing prob.** | | | |
| --- | --- | --- | --- | --- |
| *Predictors* | *Odds Ratios* | *CI* | *Statistic* | *p* |
| (Intercept) | 0.05 | 0.03 – 0.08 | -13.63 | **2.522e-42** |
| Static non-REW | 1.02 | 0.83 – 1.24 | 0.15 | 8.798e-01 |
| Dynamic REW | 12.53 | 10.57 – 14.86 | 29.08 | **6.164e-186** |
| Static REW | 12.95 | 10.92 – 15.36 | 29.47 | **6.089e-191** |
| **Random Effects** | | | | |
| σ^2^ | 3.29 | | | |
| τ_00_ _Rat_ | 0.29 | | | |
| ICC | 0.08 | | | |
| N _Rat_ | 7 | | | |
| Observations | 28 | | | |
| Marginal R^2^ / Conditional R^2^ | 0.318 / 0.374 | | | |

Pairwise comparisons

| *contrast* | *odds.ratio* | *SE* | *df* | *null* | *z.ratio* | *p* |
| --- | --- | --- | --- | --- | --- | --- |
| Dynamic non-REW vs. Static non-REW | 0.98488398 | 0.099197100 | Inf | 1 | -0.1512262 | 8.797973e-01 |
| Dynamic non-REW vs. Dynamic REW | 0.07978541 | 0.006936747 | Inf | 1 | -29.0814405 | **6.163625e-186** |
| Dynamic non-REW vs. Static REW | 0.07720730 | 0.006709095 | Inf | 1 | -29.4746281 | **6.088697e-191** |
| Static non-REW vs. Dynamic REW | 0.08100996 | 0.007011835 | Inf | 1 | -29.0356032 | **2.338755e-185** |
| Static non-REW vs. Static REW | 0.07839228 | 0.006781683 | Inf | 1 | -29.4306140 | **2.229259e-190** |
| Dynamic REW vs. Static REW | 0.96768694 | 0.066275332 | Inf | 1 | -0.4795944 | 6.315158e-01 |

S4C Fig

|  | **lever-pressing prob.** | | | |
| --- | --- | --- | --- | --- |
| *Predictors* | *Odds Ratios* | *CI* | *Statistic* | *p* |
| Intercept | 0.00 | 0.00 – 0.00 | -72.88 | **0.000e+00** |
| Static non-REW | 1.02 | 0.81 – 1.27 | 0.15 | 8.826e-01 |
| Dynamic REW | 8.88 | 7.45 – 10.58 | 24.41 | **1.474e-131** |
| Static REW | 9.05 | 7.60 – 10.78 | 24.67 | **2.153e-134** |
| time (linear) | 0.00 | 0.00 – 0.00 | -5.08 | **3.686e-07** |
| time (quadratic) | 6287165936.23 | 14052756.31 – 2812861379296.59 | 7.25 | **4.320e-13** |
| time (linear) * Static non-REW | 105.13 | 0.05 – 243641.81 | 1.18 | 2.390e-01 |
| time (linear) * Dynamic REW | 76703732.93 | 98470.75 – 59748325407.04 | 5.34 | **9.062e-08** |
| time (linear) * Static REW | 2989630.13 | 3932.61 – 2272763194.75 | 4.41 | **1.055e-05** |
| time (quadratic) * Static non-REW | 18.62 | 0.00 – 92859.36 | 0.67 | 5.009e-01 |
| time (quadratic) * Dynamic REW | 0.00 | 0.00 – 0.00 | -7.71 | **1.256e-14** |
| time (quadratic) * Static REW | 0.00 | 0.00 – 0.00 | -7.74 | **1.006e-14** |
| **Random Effects** | | | | |
| σ^2^ | 3.29 | | | |
| τ_00_ _session:Rat_ | 0.08 | | | |
| τ_00_ _Rat_ | 0.05 | | | |
| ICC | 0.04 | | | |
| N _session_ | 7 | | | |
| N _Rat_ | 7 | | | |
| Observations | 2100 | | | |
| Marginal R^2^ / Conditional R^2^ | 0.287 / 0.315 | | | |

Pairwise comparisons

| *contrast* | *degree* | *estimate* | *SE* | *df* | *z.ratio* | *p* |
| --- | --- | --- | --- | --- | --- | --- |
| Dynamic non-REW vs. Static non-REW | linear | 0.9999766 | 1.990435e-05 | Inf | -1.17591596 | 6.421035e-01 |
| Dynamic non-REW vs. Dynamic REW | linear | 0.9999080 | 1.710719e-05 | Inf | -5.37977320 | **4.462322e-07** |
| Dynamic non-REW vs. Static REW | linear | 0.9999243 | 1.704351e-05 | Inf | -4.44051793 | **5.303998e-05** |
| Static non-REW vs. Dynamic REW | linear | 0.9999314 | 1.604394e-05 | Inf | -4.27743936 | **1.112254e-04** |
| Static non-REW vs. Static REW | linear | 0.9999477 | 1.599865e-05 | Inf | -3.26753674 | **5.975216e-03** |
| Dynamic REW vs. Static REW | linear | 1.0000164 | 1.242506e-05 | Inf | 1.31595263 | 5.526891e-01 |
| Dynamic non-REW vs. Static non-REW | quadratic | 1.0000000 | 5.655230e-09 | Inf | -0.67314544 | 9.072780e-01 |
| Dynamic non-REW vs. Dynamic REW | quadratic | 1.0000000 | 4.642401e-09 | Inf | 7.71026584 | **9.903189e-14** |
| Dynamic non-REW vs. Static REW | quadratic | 1.0000000 | 4.650977e-09 | Inf | 7.73848421 | **8.437695e-14** |
| Static non-REW vs. Dynamic REW | quadratic | 1.0000000 | 4.516162e-09 | Inf | 8.76871596 | **3.097522e-14** |
| Static non-REW vs. Static REW | quadratic | 1.0000000 | 4.509876e-09 | Inf | 8.82470045 | **2.975398e-14** |
| Dynamic REW vs. Static REW | quadratic | 1.0000000 | 3.175633e-09 | Inf | 0.06214995 | 9.999139e-01 |

S4D Fig

|  | **lever-pressing prob.** | | | |
| --- | --- | --- | --- | --- |
| *Predictors* | *Estimates* | *CI* | *Statistic* | *p* |
| Static non-REW | 1.01 | 0.78 – 1.30 | 0.07 | 9.479e-01 |
| Dynamic REW | 4.01 | 3.09 – 5.22 | 5.74 | **9.438e-09** |
| Static REW | 4.24 | 3.26 – 5.51 | 6.88 | **5.816e-12** |
| Observations | 756 | | | |
| R^2^ Nagelkerke | 0.233 | | | |

Pairwise comparisons

| *contrast* | *estimate* | *SE* | *df* | *z.ratio* | *p* |
| --- | --- | --- | --- | --- | --- |
| Dynamic non-REW vs. Static non-REW | 0.9914015 | 0.13227743 | Inf | -0.06528426 | 9.999002e-01 |
| Dynamic non-REW vs. Dynamic REW | 0.2490947 | 0.24212423 | Inf | -5.74053241 | **5.655093e-08** |
| Dynamic non-REW vs. Static REW | 0.2360988 | 0.20968735 | Inf | -6.88408177 | **3.492395e-11** |
| Static non-REW vs. Dynamic REW | 0.2512551 | 0.25601899 | Inf | -5.39524970 | **4.094604e-07** |
| Static non-REW vs. Static REW | 0.2381465 | 0.22293876 | Inf | -6.43615872 | **7.350174e-10** |
| Dynamic REW vs. Static REW | 0.9478274 | 0.06323868 | Inf | -0.84731191 | 8.317742e-01 |
